# Changes in the Community Structure of Under-Ice and Open-Water Microbiomes in Urban Lakes Exposed to Road Salts

**DOI:** 10.3389/fmicb.2021.660719

**Published:** 2021-03-31

**Authors:** Isabelle B. Fournier, Connie Lovejoy, Warwick F. Vincent

**Affiliations:** ^1^Département de Biologie and Institut de Biologie Intégrative et des Systèmes (IBIS), Université Laval, Quebec City, QC, Canada; ^2^Centre for Northern Studies (CEN), Université Laval, Quebec City, QC, Canada; ^3^Québec-Océan, Université Laval, Quebec City, QC, Canada

**Keywords:** road salts, bacteria, salinization, winter, phytoplankton, protist biodiversity

## Abstract

Salinization of freshwater is increasingly observed in regions where chloride de-icing salts are applied to the roads in winter, but little is known about the effects on microbial communities. In this study, we analyzed the planktonic microbiomes of four lakes that differed in degree of urbanization, eutrophication and salinization, from an oligotrophic reference lake with no surrounding roads, to a eutrophic, salinized lake receiving runoff from a highway. We tested the hypothesis that an influence of road salts would be superimposed on the effects of season and trophic status. We evaluated the microbial community structure by 16S rRNA sequencing for Bacteria, and by four methods for eukaryotes: 16S rRNA chloroplast analysis, 18S rRNA sequencing, photosynthetic pigment analysis and microscopy. Consistent with our hypothesis, chloride and total nitrogen concentrations were among the most important statistical factors explaining the differences in taxonomic composition. These factors were positively correlated with the abundance of cryptophytes, haptophytes, and cyanobacteria. Ice-cover was also a major structuring factor, with clear differences between the winter communities and those of the open-water period. Nitrifying and methane oxidizing bacteria were more abundant in winter, suggesting the importance of anaerobic sediment processes and release of reduced compounds into the ice-covered water columns. The four methods for eukaryotic analysis provided complementary information. The 18S rRNA observations were strongly influenced by the presence of ribosome-rich ciliates, but revealed a much higher degree of taxonomic richness and greater separation of lakes, seasonal changes and potential salinity effects than the other methods.

## Introduction

In many cold temperate lakes and rivers, the use of road de-icing salts in their catchments during winter has resulted in a pronounced increase of major ion concentrations, especially of the main road salt constituents sodium and chloride (Dugan et al., 2017; Kaushal et al., 2018). This salinization effect has been shown to increase with increasing intensity of urbanization of the watershed (Dugan et al., 2017; Bird et al., 2018), and is especially apparent in winter and early spring due to the runoff from melting roadside snow (Novotny et al., 2008; Scott et al., 2019). Road-contaminated snow has a high salt content, and the subsequent meltwater inflow into lakes can cause a sudden rise in major ion concentrations; for example, salinity rose by a factor of two within a few hours of a snowmelt event in Lake Saint-Charles, Quebec (Fournier et al., 2020). These acute seasonal peaks, as well as chronic long-term salinization, have the potential to affect aquatic communities.

Most studies to assess salinization effects on freshwater biota have been done via short term (days) laboratory experiments, often at salt concentrations that are well above the usual levels of perturbation (e.g., Van Meter et al., 2011), or have been conducted by sampling over freshwater to marine salinity gradients (e.g., Herlemann et al., 2011). Smaller salinity gradients within the freshwater range have been less studied (Wallace and Biastoch, 2016), and consideration has not been given to salt impacts on freshwater microbiomes, here defined as the complete assemblage of Bacteria, Archaea, and microbial eukaryotes that underpin biological productivity, food webs and biogeochemical cycling processes (Grossart et al., 2020). Such consideration requires a focus on natural microbial communities, with their diversity of taxa and their potential responses to the presence of multiple stressors and other variables that may lessen or exacerbate the influence of salt inputs in the environment.

Given the seasonal timing of salt contamination in cold temperate lakes, analysis of salinization effects requires special attention to the overwintering microbial community. There is increasing research interest in under-ice microbiomes in lakes, and observations to date show that lakes maintain active microbial assemblages throughout winter with prokaryotic and eukaryotic communities that differ from those in summer (e.g., Kalinowska et al., 2019; Vigneron et al., 2019). The seasonal differences in metabolism and community composition of Bacteria may be due to changes in substrate availability caused by a shift in the microbial eukaryote community (Bertilsson et al., 2013), and to large winter-summer differences in physical and chemical conditions (Vigneron et al., 2019). Winter assemblages of microbial eukaryotes in different aquatic systems vary greatly (Lake Baikal, Bashenkhaeva et al., 2015; Lake Erie, Beall et al., 2016). In lakes with ice and snow cover, there appears to be a functional convergence toward dominance by phytoflagellates (chrysophytes and cryptophytes), but with large differences in species composition among different waters (Butts and Carrick, 2017). Less is known, however, about whether there is a similar taxonomic diversity with functional convergence among non-pigmented heterotrophic microbial eukaryotes (colorless protists) in winter.

Our aim in the present study was to identify the impacts of freshwater salinization on lake microbiomes, including during the less studied ice-cover period. We hypothesized that road salt exposure causes taxonomic changes in microbiome composition, and acts in concert with other known structuring factors, notably trophic state, and seasonal forcing. To assess this hypothesis, we investigated microbial community structure along with physical and chemical variables throughout all seasons, in four boreal lakes that were chosen to encompass contrasting salinity perturbations and trophic regimes.

We used four methods to analyze the community structure of the microbial eukaryotes: classic microscopy, pigment analysis by High Pressure Liquid Chromatography (HPLC), and high throughput amplicon sequencing, targeting the V4 region of 18S rRNA and additionally the 16S rRNA in chloroplasts. Sequencing of 16S rRNA amplicons was also applied to analyze the prokaryotic communities. These methods each have their own biases, strengths and weaknesses, and a secondary aim of our study was to compare these methods in evaluating taxonomic structure in the four lakes.

## Materials and Methods

### Study Sites

The four lakes selected for this study (Lake Saint-Charles, Lake Clément, Lake Saint-Augustin, and Lake Clair) are located within a 40 km-radius of Quebec City, Canada, and span a range of morphometries, trophic states, mixing regimes and other environmental conditions, including a gradient of urbanization impacts (Table 1). During the study, the mean air temperature for the ice-cover period (Jan-Feb-Mar) was –11°C, with 254 cm of snowfall. The mean air temperature for the open-water period (rest of the year) was +12°C, with rainfall totaling 751 cm (Government of Canada, 2019).

**TABLE 1:** Physico-chemical characteristics of Lake Clair, Lake Saint-Charles, Lake Clément and Lake Saint-Augustin, and the percent urbanization of their watersheds.

	Mean depth	Max depth	Max length	Max width	Area	Trophic status	Mixing type	Urbanization
	(m)				(km^2^)			%
Lake Clair	11.7	26.9	949	789	0.4	Oligotrophic	Dimictic	0
Lake Saint-Charles	4.1	17.5	5584	1248	3.6	Mesotrophic	Dimictic	8
Lake Clément	2.3	6.1	662	333	0.09	Oligotrophic	Dimictic	31
Lake Saint-Augustin	3.6	4.6	2100	300	0.6	Eutrophic	Polymictic	70*

APEL (2011, 2014, 2016), OBV de la Capitale (2018). *Estimated from Roberge (2004) and OBV de la Capitale (2018).

**FIGURE 1:**
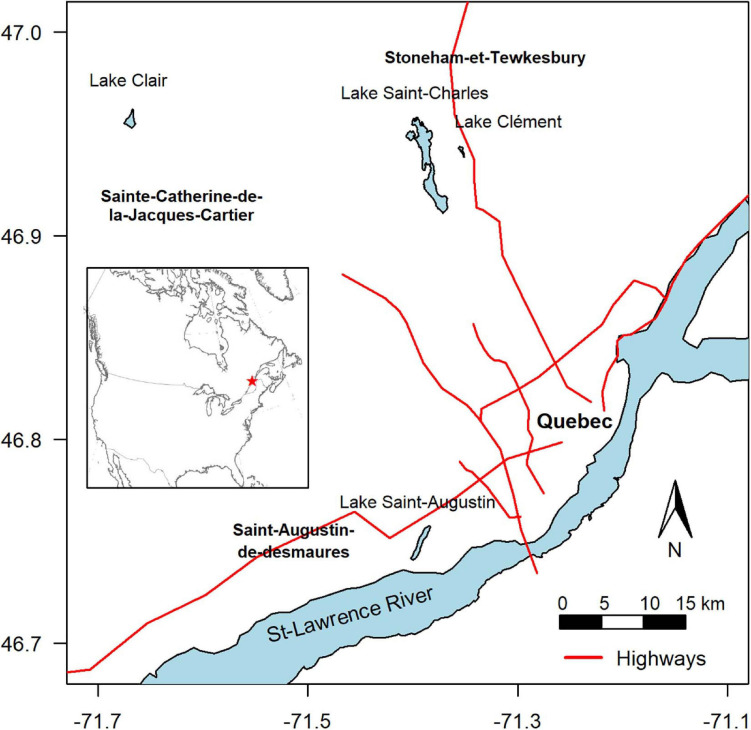
Location of the sampled lakes. The axes are degrees of latitude and longitude.

Lake Saint-Charles (LSC) is a drinking water supply for Quebec City, with the drinking water intake located 11 km downstream on its outflow, the Saint-Charles River. The lake is located in an urban area and is close to a major road (Highway 73), but most of its watershed is forested (Figure 1 and Table 1), and is considered here as a low impacted urban lake.

Lake Clément (LCL) is a recreational lake that can only be accessed by residents. It is located near Lake Saint-Charles, but closer to Highway 73, under which its main tributary flows (APEL, 2011). Its salinity is high (specific conductivity around 1000 μS cm^–1^), mainly due to the input of NaCl road salt from the highway, with chloride concentrations often exceeding the chronic exposure criterion for the protection of aquatic life of 120 mg L^–1^ (CCME, 2011). This lake is considered here as a moderately impacted urban lake.

Lake Saint-Augustin (LSA) is a recreational urban lake with public access, where motor boating and seaplane landings are permitted. It is close to a major four-lane road (Highway 40), and like Lake Clément, it has an elevated salinity as a result of road salt application. Its specific conductivity increased rapidly with the completion and full operation of the highway, from 255 μS cm^–1^ in 1970 to 700 μS cm^–1^ in 2000, with a stabilization since that time in the range 650–800 (Supplementary Figure S1). The lake has other water quality issues, including eutrophication and summer-long cyanobacterial blooms of *Aphanizomenon flos-aquae*, *Microcystis aeruginosa*, and *Dolichospermum* spp. It was included here as a heavily impacted urban lake. This lake lies in the St-Lawrence Lowlands physiographic region, while the other lakes are on the Canadian Shield; this difference in bedrock results in a higher natural background alkalinity and conductivity.

Finally, Lake Clair (LCR) is located in the Duchesnay nature reserve, and although it is in close proximity to urban areas and the other studied lakes (Figure 1), its watershed is entirely forested and without paved roads, and the lake has not experienced anthropogenic perturbations (Houle et al., 2002). It was included in the present study as a non-urban reference lake, without road salt exposure. All the lakes experience a similar climate and are covered by continuous ice and snow from December to April.

### Lake Sampling

The four lakes were sampled throughout the year between January and October 2017, except in April, when conditions were too precarious due to ice break-up, with 3 sampling dates in the under-ice period and 3–4 sampling dates in the open water period (Supplementary Table S1). Samples were taken in triplicate in a 5-m radius. During the open-water period, water was sampled at the surface (0–30 cm) directly into Aqua-Pak containers (Reliance, Manitoba, Canada) that had been acid-washed (HCl, 0.1 N), then 3 times rinsed with sample water. During the ice-cover periods, water was sampled just below the lake ice through 20 cm holes with a Kemmerer bottle, then transferred to cleaned and rinsed Aqua-Pak containers. Water from the containers was used for chemical analyses, photosynthetic pigment analyses, and characterization of the microbial communities by light microscopy. For both periods, water was also collected in sterile Nalgene bottles for RNA-based characterization of the microbial communities. Conductivity, dissolved oxygen, and temperature profiles were measured at all sampling sites with a Hydrolab DS5X profiler (Loveland, Colorado, United States).

### Chemical Analyses

Total nitrogen (TN) and total phosphorus (TP) samples were acidified (H_2_SO_4_, 0.1% final concentration), then kept at 4°C until concentrations were determined by a colorimetric method after persulfate digestion. Samples for major ions, dissolved organic carbon (DOC) and colored dissolved organic carbon (CDOM) were filtered through Milli-Q water pre-rinsed cellulose acetate filters (0.2 μm pore size, Advantec Micro Filtration Systems), then acidified (for cations only, Trace Metal Grade HNO_3_ at 0.2% final concentration) and kept at 4°C until analysis. Anion concentrations were measured by ion chromatography (ICS-2000, Dionex), major cations by atomic emission spectroscopy (ICP-AES, Varian Vista AX), and trace cations by mass spectroscopy (ICP-MS, Thermo X Series). DOC was measured by combustion catalytic oxidation (Shimadzu TOC-5000A carbon analyzer calibrated with potassium biphthalate), and CDOM was quantified by absorption at 440 nm measured in a Varian Cary 100 dual beam spectrophotometer (Varian Inc., Canada; Watanabe et al., 2011). Alkalinity (in calcium carbonate equivalents) was determined with titration by 0.01 M sulfuric acid (Wetzel and Likens, 2000), and DIC was calculated from alkalinity and the initial sample pH.

### Photosynthetic Pigment Analyses

Samples for photosynthetic pigment analyses were filtered under low light through Whatman GF/F 25 mm filters. These were then folded, wrapped in aluminum foil and stored at –80°C until extraction. The pigments were extracted for 1 h on ice in 2.7 mL of 95% methanol after sonication (3 times for 20 s at 17 W intensity). The solution was then filtered into an HPLC vial using a PTFE 0.2 μm filter. During extraction and in the HPLC vial, headspaces were filled with argon to avoid oxidation.

HPLC analyses were performed by injecting the filtrates (100 μL) into a Thermo Scientific system (Thermo Scientific, West Palm Beach, FL, United States) fitted with a Hypersil Gold C8 HPLC column (3.0 μm pore size, 4.6 × 150 mm, Thermo Scientific) at 25°C, with a C8 guard column. The pigments were eluted by a succession of polar and non-polar solvents as described in Zapata et al. (2004), and were detected by Photodiode Array (PDA) and fluorescence spectroscopy as they exited the columns; absorbance chromatograms for carotenoids were obtained at 450 nm, and fluorescence chromatograms for chlorophylls by excitation at 440 nm and emission at 650 nm. The HPLC was calibrated for 30 pigments using standards from DHI International Agency (Holshørm, Denmark) or Sigma Aldrich (Missouri, United States): 9-cis-neoxanthin, 19-but-fucoxanthin, 19-hex-fucoxanthin, alloxanthin, antheraxanthin, aphanizophyll, astaxanthin, β,β-carotene, β,ε-carotene, canthaxanthin, chlorophyll *a*, chlorophyll *b*, chlorophyll *c2*, chlorophyll *c3*, chlorophyllide *a*, crocoxanthin, diadinoxanthin, diatoxanthin, echinenone, fucoxanthin, lutein, lycopene, MgDVP, myxoxanthophyll, peridinin, pheophorbide *a*, pheophytin *a*, prasinoxanthin, violaxanthin and zeaxanthin. This calibration allowed the conversion from absorbance to concentration. For unidentified pigments with a PDA spectrum, the conversion factor of the known pigment with both the closest spectrum and retention time was used, while for those with concentrations that were too small to resolve the spectrum, β,β-carotene and chlorophyll *a* conversion factors were used for carotenoids and chlorophylls respectively. The pigments assigned to the groups “unknown chlorophylls” or “unknown carotenoids” had recognizable spectra, but they eluted apart from identified chlorophylls and carotenoids, and likely included degradation products.

The abundance of different pigments was compared in terms of molar ratios to chlorophyll *a*, with molecular weights obtained from Roy et al. (2011). For unknown pigments, the molecular weights of β,β-carotene, and chlorophyll *a* were used for carotenoids and chlorophylls, respectively. For some analyses, pigments were pooled as total carotenoids (Tot Caro: sum of known and unknown carotenoids), total chlorophylls (Tot Chl: sum of known and unknown chlorophylls, excluding degradation products of chlorophyll *a*, namely pheophytins and pheophorbides) and total chlorophyll *a* (Tot Chla: sum of chlorophyll *a* and chlorophyll *a* allomers).

A combination of 17 pigments was used to infer the proportion of the photosynthetic community associated with chlorophytes, chrysophytes, cryptophytes, cyanobacteria, diatoms, dinoflagellates, euglenophytes, and haptophytes. A Bayesian method based on CHEMTAX, and available in R (*lsei* function of the *limSolve* package), was used for these calculations (Van den Meersche et al., 2008). The input table (Supplementary Table S2) was constructed from data for freshwater species (Schluter et al., 2006), except for haptophytes for which the ratio from the CHEMTAX marine paper was used (Mackey et al., 1996).

### Microscopy

Samples for microscopy were preserved with acid Lugol’s solution (glacial acetic acid 10% v/v, potassium-iodide, and iodine; 3 μL of Lugol’s solution per mL of the sample) and stored in the dark at 4°C until analysis. Microscopy samples were analyzed using the Utermöhl protocol as described in Karlson et al. (2010). Briefly, 50–100 mL of the preserved samples were placed in a settling chamber and allowed to settle for 40–80 h, depending on the volume. The sedimented samples were examined with a Zeiss Axiovert 100 inverted microscope. The organisms were counted under visible light in phase contrast at 400X or 1000X, depending on the cell size, until 400 cells/colonies per sample were reached at each magnification.

The microbial eukaryotes were identified at the lowest taxonomic resolution possible and separated into subgroups by morphology. Biovolumes were estimated from simple geometric forms (Hillebrand et al., 1999) from photomicrographs taken through an ocular with a calibrated micrometer and the MB-Ruler software (MB-Softwaresolutions, Germany). Biovolumes were calculated from the averaged measurements of 20 specimens of cells or colonies.

### Sequencing and RNA Analysis

Samples for molecular analysis were filtered through 0.2 μm Sterivex units (Millipore), which were immediately filled with RNAlater (Life Technologies) and frozen at −80°C until extraction. The RNA was extracted using the AllPrep DNA/RNA Mini Kit (Qiagen) and the Qiagen protocol modified as follows: RLT + β-ME was injected into the Sterivex units, which were then incubated at 37°C for 45 min. Lysozyme and proteinase k were then added in the units, and they were incubated at 65°C for 15 min. In the RNA purification phase, a DNA digestion step was added between Qiagen kit steps 8 and 9: 10 μL of DNAse (Qiagen RNase free DNase set) diluted in 55 μL RDD Buffer was added in the RNeasy spin columns and left for 15 min at room temperature. RNeasy spin columns were then rinsed with Buffer RW1 as in step 8. Extracted RNA was tested for DNA contamination by a PCR and then converted to cDNA with the High Capacity cDNA Reverse Transcription Kit (Applied Biosystems-Ambion). The transcription was carried out in 20 μL (10 μL RNA template, 2 μL RT Buffer 5X, 0.8 μL dntps, 2 μL RT primers, 1 μL transcriptase and 4.2 μL water) with the following thermal cycle: 25°C for 10 min, 37°C for 120 min and 85°C for 5 min. The cDNA was stored at −80°C until further analysis.

The cDNA was amplified using two sets of primers modified with Illumina adaptors: (1) 515F/806R as modified by Apprill et al. (2015), which targets the V4 region of the 16S rRNA gene in Bacteria, Archaea and chloroplasts; and (2) 572F/1009R from Comeau et al. (2011), which targets the V4 region of the eukaryote 18S rRNA gene. PCR was carried out in a total volume of 25 μL (1 μL cDNA template, 5 μL PCR buffer (New England Biolabs), 1.25 μL reverse and forward primers, 0.5 μL mix dNTP, 0.25 μL Q5 High-Fidelity DNA Polymerase (New England Biolabs) and 15.75 μL water). Conditions of the PCR thermal cycling for each set of primers are given in Supplementary Table S3. PCR products were purified with ethanol and magnetic beads (Agencourt AMPure XP), and a second PCR was run to introduce sample tags. This reaction had an initial denaturation temperature of 98°C for 30 s, then 13 cycles of 10 s denaturation at 98°C, 30 s annealing at 55°C and 30 s elongation at 72°C followed by a final extension of 4.5 min at 72°C. The second amplicons were purified with beads as previously described, quantified with the NanoDrop 1000 (Thermo Fisher Scientific), pooled in an equimolar ratio, and then sequenced with Illumina MiSeq at the IBIS/Laval University Plate-forme d’analyses génomiques (Quebec City, QC).

Forward and reverse read pairs were merged using BBMerge v37.36 (Bushnell et al., 2017), and the obtained merged reads were filtered with maximum expected error of 1 (Rognes et al., 2016). Reads with the same sequences were identified as well as abundance- and size-filtered to discard potential chimera (>300 bp) and singletons using VSEARCH (Rognes et al., 2016). UPARSE was then used to cluster reads for Operational Taxonomic Units (OTUs) at 98% similarity level for 18S rRNA and at 97% similarity level for 16S rRNA (Edgar, 2013). We used mothur v1.39 (Schloss et al., 2009) to assign the taxonomy of the most abundant sequence of each OTU based on the Protist Ribosomal Reference database (PR2; Guillou et al., 2013) for 18S rRNA and SILVA 132 (Quast et al., 2013) for 16S rRNA. OTU tables were constructed with the number of reads per OTU in each sample.

The 18S rRNA sequences identified as embryophytes, arthropods, rotifers and mitochondria were removed from further analysis. A separate analysis was made of chloroplast reads from the 16S rRNA data. When pertinent, unidentified OTUs were submitted to a BLAST search to the nr database of NCBI GenBank and identified to the closest match. All the nucleotide sequence data are available in the NCBI database under the project number PRJNA681583.

### Statistical Analyses

The dissimilarities between samples were visualized using non-metric multidimensional scaling (NMDS) analysis, with the *metaMDS* function of the *vegan* package (v2.5-6, Oksanen et al., 2019). The role of different explanatory variables in the 16S rRNA, chloroplast 16S rRNA, 18S rRNA (reads pooled at the OTU level) and microscopy analyses, was evaluated with distance-based redundancy analysis (dbRDA) and partial dbRDA. The dbRDA computes the overall variation among the communities that could be explained by the provided explanatory variables, while the partial dbRDA calculates the contribution of those explanatory variables individually. These analyses are similar to redundancy analysis but allow for non-Euclidean distances (Legendre and Anderson, 1999). In the present study, they were carried out using the *capscale* function of the *vegan* package, using Bray-Curtis distance on abundances after a Hellinger transformation. Among all measured physico-chemical parameters, those included in the dbRDA model as explanatory variables were selected by the stepwise model building function *ordistep*, from the *vegan* package. The selected explanatory variables were: Al, DIC, Fe, lake, Cl, period, Si, TN, and TP. The final analysis was conducted by period, so this variable was removed, and we kept only variables that had a correlation of less than 0.6 with one another; DIC and Fe were therefore removed because of correlation with Cl and Al, respectively.

The partial dbRDA and the NMDS permitted us to identify variables that explained the greatest variation between the samples. The organisms that responded the most to those variables were discriminated using DESeq to detect differential abundances, using the *DESeq2* package (v1.26.0, Love et al., 2014), which normalized the samples to correct for the difference in sequencing depth. The difference in abundance between the levels of the variables was then calculated as the log base 2 of the fold change. The OTUs that contributed significantly to the difference between groups were plotted on a heatmap, using the *ComplexHeatmap* package (v2.3.4, Gu, 2016), where each OTU (column) and each combination of seasonal period and lake (rows) were sorted using hierarchical clustering with the *dendsort* function of the *dendsort* package (v0.3.3, Sakai et al., 2015). The same method was also applied to microscopy data, using the lowest taxonomic ranks.

Diversity was evaluated by the Shannon index using the *diversity* function of the *vegan* package, and evenness was evaluated by the Pielou index, which was calculated as the Shannon index divided by the natural logarithm of the number of OTUs/species in a given sample. For diversity and evenness analyses, the 16S rRNA, chloroplast 16S rRNA and 18S rRNA reads data sets were rarefied, to respectively 7000, 550, and 2000 reads per sample, using the *rarefy* function of the *vegan* package to account for differences in sequencing depth between samples.

The effect of the groups on various variables was determined by a Student’s *t*-test followed by a *post hoc* Dunn test with Bonferroni correction on the *p*-value using, respectively, the *t.test* function of the *stats* base package and the *dunn.test* function of the *dunn.test* package (v1.3.5, Dinno, 2017). To evaluate the effect of groups on community composition, we used non-parametric multivariate analysis of variance (PERMANOVA) followed by a pairwise *post hoc* test using, respectively, the *adonis* function of the *vegan* package and the *pairwise.adonis* function from the *pairwiseAdonis* package (v0.0.1, Martinez Arbizu, 2020). To evaluate the relationship between various parameters, and statistical significance, linear models were constructed using the *lm* function of the *stats* base package. Differences in distribution were evaluated with Pearson chi-square test using the *chisq.test* function of the *stats* base package. All statistical analyses were executed in R (v3.6.3, R Core Team, 2013). Environmental data and analyses are available on GitHub at: https://github.com/isaza233/Road_salts_lakes_microbiome.

## Results

### Lake Chemistry

**FIGURE 2:**
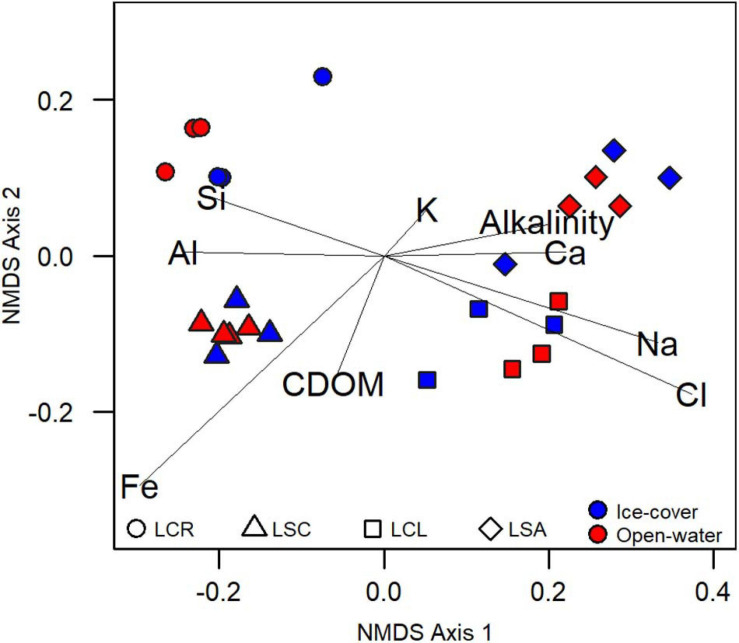
Non-metric multidimensional scaling analysis of samples based on chemical variables. Axis 1 is associated with alkalinity, conductivity, Ca, Cl, Alkalinity, K, and Na (linear models *R*^2^ > 0.75; *p* < 0.05), while Axis 2 is weakly associated with Fe and CDOM (R^2^ from linear models are < 0.4; *p* < 0.05).

The four lakes differed greatly in their chemical properties. The 25 samples clustered into four water chemistry groups that separated according to lake (ANOVA, *p* < 0.05; NMDS, Figure 2), and there was no distinction between the ice-cover and the open-water periods (ANOVA, *p* > 0.05). The first axis was correlated with alkalinity, conductivity, Ca, K, Na, and Cl (linear models *R*^2^ > 0.75; *p* #x003C; 0.05), while the second axis was only weakly correlated with Fe and CDOM (R^2^ from linear models < 0.5; *p* < 0.05). In this ordination, the lakes remained distinct and separated throughout the year, and the alkalinity/salinity gradient on the first axis also persisted throughout the year.

The chemical variation between the sampled lakes was mainly due to the concentrations and relative proportions of Ca, Cl, and Na (Table 2). The latter ions were 3–50 times lower in Lake Clair compared to Lake Saint-Charles and 20–400 times lower than in the other two lakes. In Lake Clair, Ca was the dominant cation, and Cl concentrations was <1.5 mg L^–1^, while in the other lakes, Na was the dominant cation, and Cl concentrations varied between 10 and 183 mg L^–1^, reflecting their contamination by road salts (Mochizuki, 2011; Guesdon et al., 2016; Fournier et al., 2020). As expected, the highest concentrations of TP were found in Lake Saint-Augustin (14–134 μg L^–1^), and the lowest concentrations of TN and TP were found in Lake Clair (respectively, 0.1–0.2 mg L^–1^ and 4–10 μg L^–1^).

**TABLE 2:** Mean (coefficient of variation, SD as % mean) of chemical variables in surface water (0–30 cm) for Lake Clair, Lake Saint-Charles, Lake Clément, and Lake Saint-Augustin during ice-cover (Jan–Feb–Mar) and open-water (rest of the year) periods.

Variable	Units	Lake Clair				Lake Saint-Charles				Lake Clément				Lake Saint-Augustin			
		Ice-cover		Open-water		Ice-cover		Open-water		Ice-cover		Open-water		Ice-cover		Open-water	
Conductivity	μS cm^–1^	19	(0)	17	(3)	110	(24)	77	(14)	694	(45)	707	(9)	624	(5)	644	(3)
Alkalinity	mg CaCO_3_ L^–1^	8	(9)	7	(5)	17	(26)	15	(8)	66	(18)	71	(30)	114	(9)	117	(28)
CDOM	m^−1^	1	(89)	1	(86)	13	(23)	17	(14)	16	(19)	10	(9)	18	(36)	10	(91)
Ca	mg L^–1^	2	(9)	2	(1)	7	(12)	6	(18)	21	(22)	29	(12)	40	(26)	43	(13)
Cl	mg L^–1^	0.4	(32)	0.6	(92)	19	(45)	13	(16)	77	(15)	149	(17)	86	(22)	112	(2)
DIC	mg L^–1^	3	(5)	3	(18)	5	(4)	4	(18)	18	(27)	18	(13)	31	(27)	29	(6)
Na	mg L^–1^	1	(7)	1	(27)	11	(34)	7	(16)	50	(22)	87	(16)	57	(21)	70	(2)
Si	mg L^–1^	1.3	(3)	1.3	(3)	3.4	(4)	2.4	(10)	2.2	(23)	1.4	(18)	2.0	(27)	2.4	(33)
TN	mg L^–1^	0.2	(18)	0.1	(8)	0.6	(11)	0.4	(15)	0.7	(7)	0.4	(6)	0.7	(23)	0.7	(35)
Al	μg L^–1^	11	(9)	17	(39)	48	(13)	43	(29)	18	(18)	32	(32)	14	(81)	31	(25)
Fe	μg L^–1^	13	(4)	2	(102)	178	(9)	156	(35)	86	(19)	58	(95)	14	(72)	7	(51)
TP	μg L^–1^	6	(42)	8	(25)	9	(18)	16	(29)	11	(171)	8	(46)	20	(28)	79	(56)

*DIC, Dissolved inorganic carbon; TN, total nitrogen; TP, total phosphorus; CDOM, colored dissolved organic carbon.*

### Water Column Profiles

All lakes exhibited similar year-round patterns of temperature with warmer water during the open-water period and the usual inverse stratification profiles (cold overlying warmer water) during the ice-cover period (Supplementary Figure S2). A thermocline was observed during most of the open-water period, except during spring and autumn overturn.

Oxygen in the surface water was near saturation with the atmosphere throughout the year in all lakes, while in the bottom water, oxygen levels were often well below saturation (≈50%), including periods of anoxia during summer/autumn in Lake Saint-Augustin and Lake Clément (Supplementary Figure S2).

Salinity, as measured by specific conductivity, was the most variable parameter, whether by depth or among lakes (Supplementary Figure S2). Lake Clair exhibited minor fluctuations in conductivity down the water column and over the year (in the range 17–22 μS cm^–1^), with small increases in bottom water during the open-water period. Lake Saint-Charles conductivity was about 10-times greater than Lake Clair, and always around 50% greater in surface compared to bottom waters. Conversely, in Lake Clément and Lake Saint-Augustin, conductivity was always 2–4 times greater in the bottom water, consistent with these lakes being fed by denser, saltier runoff from the roads. Also, the conductivities of the two lakes were 3–13 times higher than in Lake Saint-Charles. In these three lakes, maximum conductivity was observed during the late summer period of lowest inflows and the winter period of road salt use.

For the chemical and the biological analyses, all water was sampled from the surface, and the sampled communities from the four lakes were therefore from similar temperature and oxygenation levels for any period of sample collection, but with large differences in conductivity among sites (<25–1,000 μS cm^–1^).

### Microbiome Composition

**FIGURE 3:**
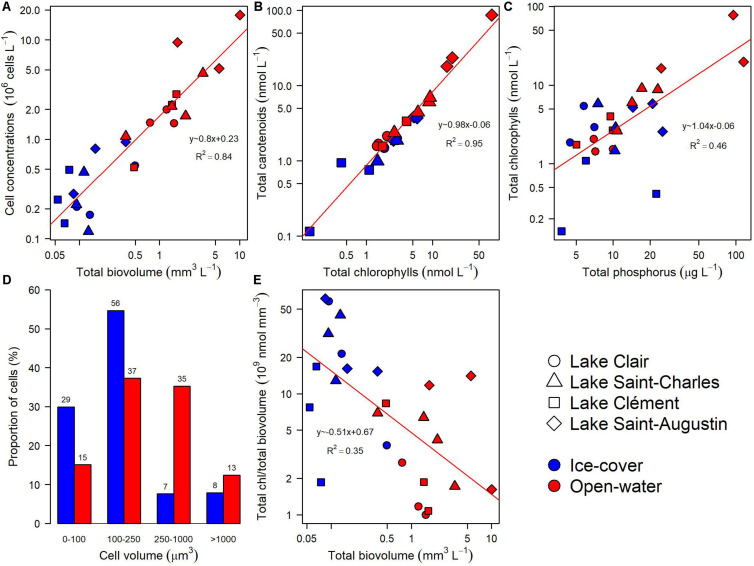
General relationships for the microbial eukaryote community from Lake Clair, Lake Saint-Charles, Lake Clément, and Lake Saint-Augustin during the ice-cover and the open-water periods based on microscopy and photosynthetic pigment analyses. **(A)** Cell concentration versus total biovolume. **(B)** Total carotenoids versus total chlorophylls. **(C)** Total chlorophylls versus total phosphorus. **(D)** Proportion of cells in four size classes. **(E)** Total chlorophylls per unit biovolume versus total biovolume.

The five identification methods provided complementary information on the taxonomic composition of the microbial community. During the ice-cover period, there was a selection for smaller cells with a higher chlorophyll content than in the open-water period, and the overall cell abundance was lower (Figures 3A,D,E). In both periods, the total carotenoids were correlated with total chlorophylls (Figure 3B, *R*^2^ = 0.95). The chlorophyll concentration was correlated with total phosphorus when all samples were considered, but when samples from different periods were analyzed separately, this relationship was only significant during the open-water period (Figure 3C, *R*^2^ = 0.82).

The 16S rRNA primers yielded 1,079,370 non-chloroplast reads, with a mean of 25 101 per sample. They were grouped in 711 OTUs, of which 97% were identified at the phylum level, 38% at the genus level, and only 5% at the species level. The communities were dominated by Proteobacteria (44–69%), represented mainly by the classes Betaproteobacteria (24–47%) and Alphaproteobacteria (9–27%) (Supplementary Figure S3). At the genus level there was more variation between lakes and periods, but some common genera emerged including *Polynucleobacter, Sediminibacterium, Polaromonas, Rhodoferax*, and *Caulobacter* (Supplementary Tables S4–S11).

We obtained 337,290 chloroplast reads from the 16S rRNA amplicons, that were grouped in 137 OTUs, with a mean of 7,844 reads per sample. The majority of the OTUs were identified at the phylum level (91%), 77% were identified at the genus level, and 72% at the species level. Depending on the lake, communities were either dominated by chrysophytes (2–79%) or cryptophytes (2–44% and Supplementary Figure S4). The most abundant genera were *Synura, Cryptomonas*, and *Florenciella* (Supplementary Tables S4–S11).

The 18S rRNA primers yielded 251,390 reads, with a mean of 5,982 reads per sample. They were placed into 812 OTUs, of which 97% were identified at the phylum level, 29% at the genus level and 10% at the species level. The dominant phylum differed between lakes and periods. The three most abundant phyla were Ochrophyta (2–40%, mostly chrysophytes except for 1 OTU affiliated to the diatom *Stephanodiscus*), cryptophytes (2–38%) and ciliates (2–55%, Supplementary Figure S3). In these groups, *Dinobryon, Synura, Cryptomonas, Mesodinium*, and ciliates from the Class Spirotrichae were common and abundant (Supplementary Tables S4–S11).

Among all lakes and the two periods, a total of 79 different microorganisms were observed by inverted microscopy, 78% of which were identified at the genus level and 22% at the species level. The communities were dominated by chlorophytes (2–38% by biovolume) and chrysophytes (0–64% by biovolume, Supplementary Figure S5). *Strobilidium, Dinobryon, Cryptomonas*, and *Chlamydomonas* were the most common genera (Supplementary Tables S4–S11).

The HPLC analysis detected 159 photosynthetic pigments, with a mean of 35 pigments per sample. The number of chlorophylls and carotenoids were similar (respectively, 43 and 57%). Some pigments were identified by comparison to the standards (19%) or related to them (similar spectrum but different retention time, 21%), while the rest had different spectra and retention times, and could not be identified. The most abundant pigments were chlorophyll *a*, *c1*, and *c2*, fucoxanthin, alloxanthin, and diadinoxanthin (data not shown). The communities, based on the composition inferred by the ratio of 17 selected pigments, were dominated by chrysophytes (13–79%), dinoflagellates (0–62%), and cryptophytes (6–46%, Supplementary Figure S4).

Although the four methods used to identify the microbial eukaryotes were consistent at the group and even genus levels, there were also major differences. Pigments associated with diatoms were detected, and diatoms were observed by microscopy (e.g., *Asterionella, Tabellaria*, and *Synedra*) and chloroplast 16S rRNA (e.g., *Acanthoceras* and *Skeletonema*) in half or more of the samples, while only 1 OTU affiliated with this group in one sample was identified by 18S rRNA (*Stephanodiscus*, Supplementary Figure S4). The analysis of photosynthetic pigments revealed the presence of euglenophytes. These were also observed in microscopic analysis of live samples from 10 μm plankton net hauls (unpublished observations), but they were rare in the microscopic counts of preserved samples and not detected at all by the molecular analysis, neither as chloroplast 16S rRNA nor 18S rRNA (Supplementary Figure S4). More chlorophytes were identified with microscopy than with any other method (Supplementary Figure S4). The chloroplast 16S rRNA analysis was the method that identified the least number of different phototrophs with no detection of dinoflagellates, euglenophytes, or chlorophytes (Supplementary Figure S4). The 18S rRNA analyses detected many more ciliates than microscopy (Supplementary Figure S3 vs. Supplementary Figure S5). For example, in Lake Clair during the ice-cover period, *Mesodinium* made up 40% of the reads, while in microscopy, it represented only 0.4% of the biovolume (Supplementary Table S5).

## Identification of Environmental Drivers

**FIGURE 4:**
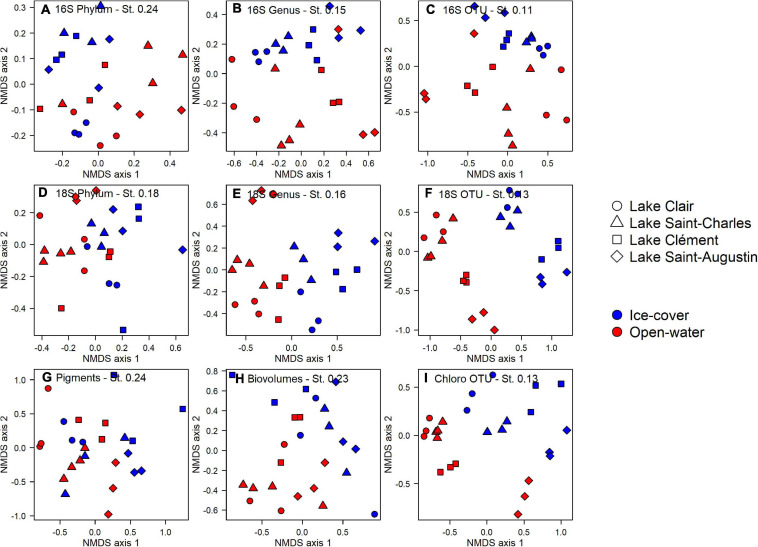
Non-metric multidimensional scaling analysis of microbial community structure in the surface waters of Lake Clair, Lake Saint-Charles, Lake Clément, and Lake Saint-Augustin for the ice-cover and the open-water periods. Sequences from 16S rRNA and 18S rRNA pooled at the phylum **(A,D)**, genus **(B,E)** and OTU **(C,F)** levels, **(G)** pigment concentrations, **(H)** microscopy biovolumes, and **(I)** chloroplast 16S rRNA reads pooled at OTU level. St NMDS stress.

At the phylum level for the prokaryotes, there was no differentiation between lakes and periods (PERMANOVA, *p* > 0.05, Figure 4A). However at the genus and the OTU levels, the lakes and periods were clearly separated (PERMANOVA, *p* < 0.01, Figures 4B,C). A pairwise *post hoc* test further identified Lake Clair and Lake Saint-Charles as being similar at the OTU level, the two lakes with the lowest salinization, while Lake Clément and Lake Saint-Augustin were different from them and from each other (*post hoc* test, *p* < 0.01). Despite these distinctions, most of the OTUs were shared between the ice-cover and the open-water periods (77%); of the OTUs found only during a given period, 5% were from the ice-cover period and 95% from the open-water period. Almost half of the OTUs (40%) were shared among all the lakes, with few found only in either low or high conductivity lakes (respectively, 8 and 3%). The Shannon diversity indices were around 4, and neither this metric nor evenness (around 0.75) of the bacterial communities showed an influence of period (ice-cover vs. open-water) or salinity (*t*–test, *p* > 0.05).

The photosynthetic pigments (based on the 159 detected pigments) indicated an effect of season (PERMANOVA, *p* = 0.037) and lakes (PERMANOVA, *p* < 0.01, Figure 4G), but for the latter, it was only between Lake Clair and Lake Saint-Augustin, the two extremes of the salinization/urbanization gradient. Only a small proportion of the pigments were detected in all the lakes (23%), and a smaller proportion, respectively, 5 and <1%, were uniquely found in the highest and the lowest salinization lakes (data not shown). About half of the pigments were detected year-round, and among those detected only in one period, the majority (89%) were detected in the open-water period. The pigments detected exclusively during the ice-cover period were chlorophylls and carotenoids (or their breakdown products) that could not be fully identified by the standards.

The microscopy-derived biovolume data showed significant differences between the two periods (PERMANOVA, *p* < 0.01), but not among lakes (Figure 4H). The diversity of the community was higher during the open-water period (*t*-test, *p* < 0.01, 1.81 vs. 1.14), but there was no difference in evenness. The diversity index was about two times lower when calculated from microscopy than from the chloroplast 16S rRNA or 18S rRNA. A notable proportion of species were shared between lakes (22%) and periods (40%). No species was unique to the lakes with the highest salinization, while 13% of the species were only found in the lowest salinization lakes. There were unique species observed in the open-water period (53%), while few were unique to the ice-cover period (7%).

The photosynthetic eukaryotic community inferred from the chloroplast 16S rRNA sequences showed significant differences between periods and among the lakes (PERMANOVA, *p* < 0.01, Figure 4I). The lakes with the lowest salinization, Lake Clair and Lake Saint-Charles, were different from Lake Saint-Augustin, the lake with the highest salinization (*post hoc* test, *p* < 0.01). The diversity was higher during the open-water period (*t*-test, *p* < 0.01, 3.09 vs. 1.85), while the evenness was higher during the ice-cover period (*t*-test, *p* < 0.01, 0.97 vs. 0.78). The majority of OTUs were shared between the two periods (79%, data not shown), while only a small proportion of the OTUs were shared among all lakes (35%, data not shown). Concerning the OTUs that were only found in one period, the majority were unique to the open-water (93%, data not shown). No OTU was unique to the lakes with the highest salinization, while 15% of the OTUs were only found in the lakes with the lower salinization.

The 18S rRNA analyses showed differences between the ice-cover and the open-water at the phylum, the genus, and the OTU levels (PERMANOVA, *p* < 0.01, Figures 4D–F). The effect of lake site was only apparent at the genus and the OTU levels (PERMANOVA, *p* < 0.01, Figures 4E,F); a pairwise *post hoc* test revealed that, at the OTU level, Lake Clair and Lake Saint-Charles were similar, and Lake Clément and Lake Saint-Augustin were similar, but the two types of lakes differed (*p* < 0.01), with only 17% of OTUs shared by all lakes. A small proportion of the OTUs were unique to the lakes with the higher salinization (3%), while 19% of the OTUs were found only in the lakes with the lower salinization. About half of the OTUs were shared between the two periods, while 13% were unique to the ice-cover period, and 40% were exclusive to the open-water. The 18S rRNA communities were more diverse (*t*-test, *p* < 0.01, 4.11 vs. 3.14) and more even (*t*-test, *p* < 0.01, 0.79 vs. 0.67) during the open-water period.

**FIGURE 5:**
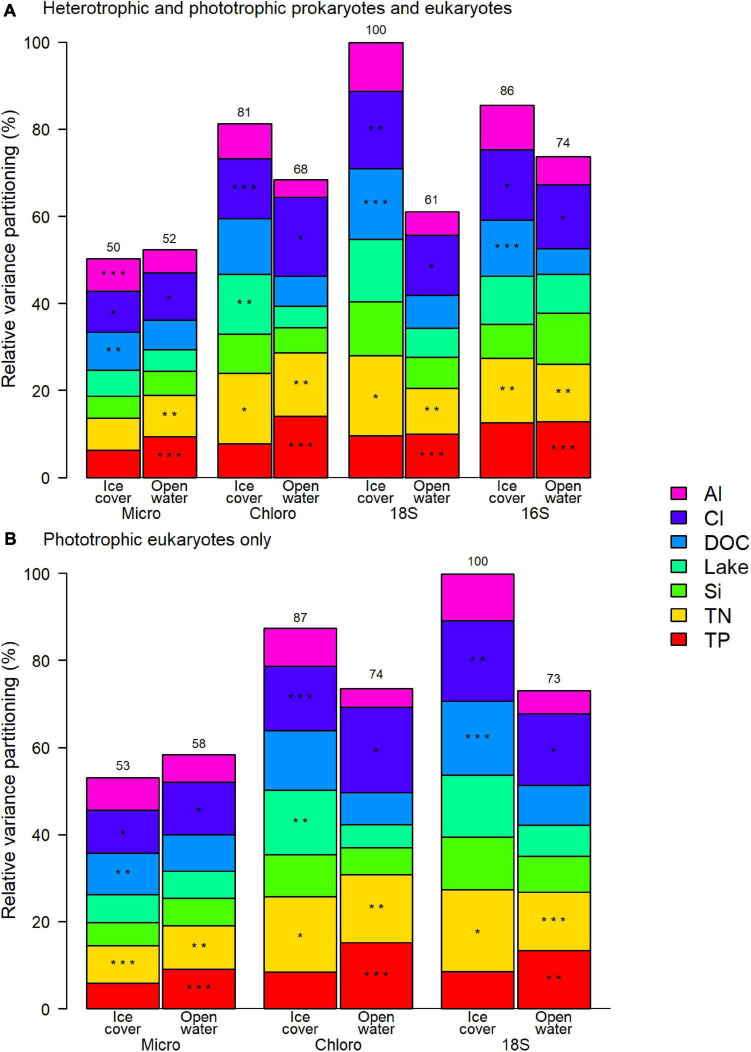
Relative contribution to the variance of selected physico-chemical factors for **(A)** the entire prokaryote and eukaryote communities, and **(B)** phototrophic eukaryotes. Asterisks represent the order of the three most important factors and the number over the column is the total variance explained by the model relative to the highest explained variance, which was for 18S ice-cover in both panels. Micro microscopy biovolumes; Chloro chloroplast 16S rRNA reads; 18S 18S rRNA reads; 16S 16S rRNA reads.

The variation of the microbial community was correlated in similar proportion to the selected factors for both prokaryotes and eukaryotes, with all applied methods (Figure 5A). The relative importance of each selected factor remained the same when only the phototrophic eukaryotes were considered (Figure 5B). For both periods, the two most consistently important factors were chloride (Cl) and total nitrogen (TN) concentrations.

### Taxonomic Responses to Environmental Drivers

Data from the two methods with the highest resolution in the separation of the samples (16S rRNA and 18S rRNA) were further used to identify changes in the abundance of taxonomic groups in response to the salinization gradient and ice-cover vs. open-water periods; for the microbial eukaryotes, the results from the microscopy and chloroplast 16S rRNA were similar to those from 18S rRNA and are presented in Supplementary Material (Supplementary Tables S12, S13).

**FIGURE 6:**
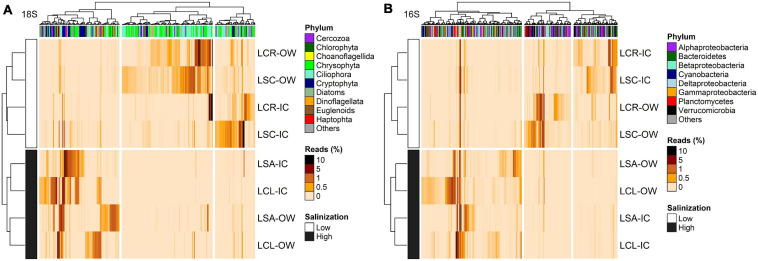
Heatmap of the abundance of OTUs (columns) identified by DESeq to discriminate between the lakes with lowest (LCR and LSC) and highest (LCL and LSA) salinization levels for **(A)** 18S rRNA (*n* = 368, *p* < 0.05) and **(B)** 16S rRNA (*n* = 382, *p* < 0.05). Phylum to which these OTUs are affiliated is displayed. The analysis was conducted on the two periods (ice-cover and open-water) combined. Rows and columns are ordered by hierarchical clustering. LCR Lake Clair; LSC Lake Saint-Charles; LCL Lake Clément; LSA Lake Saint-Augustin; IC ice-cover period; OW open-water period.

**FIGURE 7:**
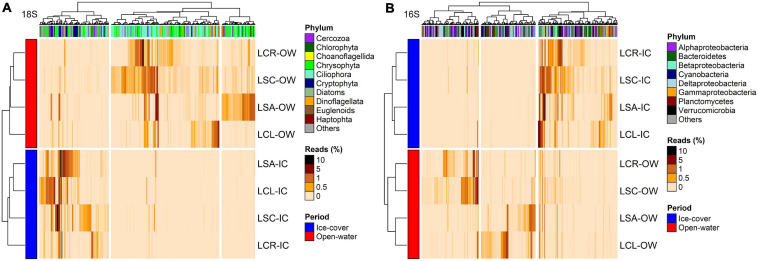
Heatmap of the abundance of OTUs (columns) identified by DESeq to discriminate between the ice-cover (Jan–Feb–Mar) and the open-water periods for **(A)** 18S rRNA (*n* = 394, *p* < 0.05) and **(B)** 16S rRNA (*n* = 393, *p* < 0.05). Phylum to which these OTUs are affiliated is displayed. The analysis was conducted on the four lakes combined. Rows and columns are ordered by hierarchical clustering. LCR Lake Clair; LSC Lake Saint-Charles; LCL Lake Clément; LSA Lake Saint-Augustin; IC Ice-cover period; OW Open-water period.

For the 18S rRNA, 368 OTUs were identified as differentially abundant between the lakes with the lowest (Lake Clair and Lake Saint-Charles) and the highest (Lake Clément and Lake Saint-Augustin) urbanization levels (Figure 6A). The lakes with the lowest salinization and urbanization levels were characterized by chrysophytes and ciliates, mainly *Dinobryon sociale* and *Mesodinium* sp. The other group of lakes was characterized by the increase in the abundance of dinoflagellates, the cryptophytes *Plagioselmis* and *Cryptomonas*, and the haptophyte *Diacronema* (Figure 6A and Supplementary Table S12). A second DESeq analysis was conducted to identify OTUs (*n* = 394) that discriminated between the ice-cover and open-water periods (Figure 7A). The ice-cover period was characterized by chrysophytes, ciliates, and cryptophytes, with the more influential being *Synura* sp., and spirotrich ciliates (Supplementary Table S13). During the open-water period, there were also more chrysophytes and ciliates, mainly *Dinobryon* sp., and Litostomatea ciliates (Figure 7A and Supplementary Table S13).

For the 16S rRNA analyses, certain OTUs differed between the two sets of lakes (Lake Clair and Lake Saint-Charles vs. Lake Clément and Lake Saint-Augustin, Figure 6B). The most prominent OTUs in the least salinized lakes were identified as Verrucomicrobia *Chthoniobacter* sp. and soil-related Bacteroidetes (Supplementary Table S12). The lakes with the highest salinization were characterized by an increase in Planctomycetes, Bacteroidetes and cyanobacteria such as *Algoriphagus hongiella* and *Cuspidothrix* LMECYA-163 (Supplementary Table S12). A further DESeq analysis identified OTUs that were differentially abundant between the ice-cover and the open-water periods (Figure 7B). For both periods, these OTUs were mainly represented by Proteobacteria and Bacteroidetes, with *Methylobacter* sp., *Nitrosospira* sp., and *Flavobacterium* sp. showing the largest increase in abundance during the ice-cover period, and among the OTUs that increase the most in abundance during the open-water period most were unclassified at the genus level, but some were identified as *Dinghuibacter* sp. and *Rhizorhapis* sp. (Supplementary Table S13). An increase in the abundance of reads Deltaproteobacteria and cyanobacteria were almost exclusively seen during the open-water period (Figure 7B). Hierarchical clustering analysis reinforced the similarity of Lake Clair and Lake Saint-Charles, and of Lake Clément and Lake Saint-Augustin (Figure 7B).

## Discussion

In this study, we analyzed the seasonal microbiomes of four lakes across a gradient of exposure to road salts. The results revealed that the taxonomic composition varied with degree of salinization (from unimpacted to strongly contaminated by road salts) in both the ice-cover and the open-water periods. The variables related to salinization, such as Cl concentration, were among the most explanatory statistical factors, along with total nitrogen (TN), independent of the period and the identification method. For the eukaryotes, all methods resulted in the identification of the same factors with similar proportions of the total variation, but the depiction of community composition and its associated changes differed by method.

### Identification of Environmental Drivers

During both periods, the concentration of chloride, and the concentration of total nitrogen were the most important drivers of the taxonomic composition of the microbial communities, both prokaryotes and eukaryotes, with all methods. In the studied lakes, Cl concentration is a proxy for total salinity, which is mostly influenced by bedrock type and urbanization level, as well as by the related road salt use. It was also correlated with alkalinity, which has previously been observed in road salt contaminated freshwater and attributed to concrete pavement weathering and cation exchange (O’brien and Majewaki, 1975; Moore et al., 2017). Sodium ions from road salts are exchanged with other major ions in soils, including calcium, which would contribute to the increased alkalinity (Shanley, 1994). This phenomenon likely occurred in the urbanized lakes as the sodium to chloride ratio was <1 (in the 0.5–0.7 range), while it would have otherwise been at least 1 due to the main source of sodium and chloride from road salt NaCl. Total nitrogen was not correlated with chloride, but its concentration was still higher in the three more urbanized lakes, compared to reference Lake Clair. The results suggest that the two most important drivers of taxonomic composition were related to the urbanization level of the watersheds.

While salinity is known to be a structuring factor for microbial communities across freshwater, brackish and marine environments, the results of this study are among the few providing evidence that there might also be an effect of salinity (or an unknown correlate of salinity) between the lowest and the highest ends of the freshwater range (Butts and Carrick, 2017; Toporowska et al., 2018). For Bacteria, the increase in salinity that occurs in the transition from a freshwater to a marine ecosystem, generally leads to a decrease in abundance of Actinobacteria, Verrucomicrobia and Betaproteobacteria and an increase in abundance of Alpha-, and Gammaproteobacteria (Bouvier and del Giorgio, 2002; Herlemann et al., 2011; Eiler et al., 2014). This switch of phylum dominance was not observed in the present study, most likely because the salinity range was too small, but two representatives of the Gammaproteobacteria were identified as having a higher abundance in the lakes with the higher salinity. For cyanobacteria, the most well-known genus with halotolerant species is *Synechococccus*, but it was not detected in the present study (Celepli et al., 2017). However, the filamentous cyanobacterium *Dolichospermum* was detected, and is known to include halotolerant strains, for example in the Baltic Sea (Celepli et al., 2017). The response of eukaryotes was more pronounced, consistent with previous observations that bacteria are generally less responsive to changes in salinity than eukaryotes (Casamayor et al., 2002). Chrysophytes and ciliates were more abundant in the lakes with the lower salinity, while cryptophytes and haptophytes were more characteristic of lakes of higher salinity. Taxa with the highest increase in abundance between these two sets of lakes include halotolerant genera. The haptophyte *Diacronema* is frequently observed in brackish and marine environments (Balzano et al., 2015). The haptophyte *Chrysochromulina* was found to increase along a transect from a river mouth to the sea (Bazin et al., 2014). The cryptophyte *Plagioselmis*, along with other cryptophytes, were observed to be the dominant components of a phytoplankton bloom in an estuary with a salinity of 20 ppt (Bazin et al., 2014). Cryptophyte abundance was also found to increase with salinity in a microcosm experiment from freshwater to 51 ppt (Greenwald and Hurlbert, 1993), and to be positively correlated with conductivity in freshwater lakes (Toporowska et al., 2018). A freshwater strain of the *Chroomonas* clade grew at osmolalities as high as 420 mOsmol kg^–1^ (equivalent to 15 ppt; Saoud et al., 2007; Hoef-Emden, 2014).

Urbanization is often associated with eutrophication, and species characteristic of urban lakes are therefore often bloom-forming cyanobacteria (Napiórkowska-Krzebietke and Dunalska, 2015; Lévesque et al., 2020). Anthropogenic factors such as logging and pasture development can also select for specific phytoplankton traits (e.g., pigment composition in relation to N:P ratio and heterotrophy; Loewen et al., 2021). Other species known in urban lakes include dinoflagellates, and diatoms such as *Cyclotella* and *Stephanodiscus*, which were identified as constituents in the present study (Napiórkowska-Krzebietke and Dunalska, 2015). The bloom-forming cyanobacterium *Dolichospermum* was found in the most urbanized lakes in the present study, but this response was mainly driven by its high abundance in hyper-eutrophic Lake Saint-Augustin. However, urbanized Lake Clément has no eutrophication problem, nor a higher abundance of cyanobacteria, yet the composition of its microbial community was more similar to highly urbanized and hyper-eutrophic Lake Saint-Augustin, than to less eutrophic, but also less urbanized Lake Clair and Lake Saint-Charles. This indicates the importance of other factors related to urbanization, such as the road salts, or of other common contaminants of urban watersheds including pharmaceuticals, toxins and metals from wastewater, sewage and roads (Hong et al., 2018; Ondarza et al., 2019; Dos Santos et al., 2020; Tian et al., 2020). While the exact factor, or combination of factors, to which they respond remain uncertain, the results of the present study suggest that an increase in abundance of bloom-forming cyanobacteria, cryptophytes or haptophytes is exacerbated by urbanization and the associated road salt effects.

### The Structuring Effect of Ice-Cover

The presence of an ice-cover leads, directly or indirectly, to many physical (light, temperature, exchange with atmosphere, wind-driven mixing), chemical (redox gradients, dissolved gas concentrations) and ecological (predator populations) changes that result in a markedly different environment than in the open-water period. The distinct communities observed in the present study between the ice-cover and the open-water periods, are likely a complex response to this combination of differences. In the Saint-Charles River, ice-cover was identified as a major driving factor of community composition for both prokaryotes (Cruaud et al., 2019a) and eukaryotes (Cruaud et al., 2019b). As in the present study, phytoplankton and ciliate communities sampled year-round in three lakes in Poland did not cluster according to the four seasons, but rather by winter (ice-cover) and the rest of the year (Kalinowska et al., 2019). In Lake Erie, the expanse of ice cover during winter was also a main segregating factor for both Bacteria and microbial eukaryote communities (Beall et al., 2016).

The bacterial communities during the ice-cover period appeared to have distinctive biogeochemical functions. In the present study, *Nitrospira* and *Nitrotoga* were identified as more abundant during the ice-cover than the open-water period. These nitrifying bacteria are often found together in wastewater treatment plants, and *Nitrotoga* has been identified as a low-temperature adapted genus (Wegen et al., 2019). Their greater abundance during winter is consistent with reports drawing attention to the importance of nitrification in lakes under winter ice cover (Cavaliere and Baulch, 2019; Massé et al., 2019). Another genus that was more abundant in the ice-cover period was *Methylobacter*, a methane oxidizer typically found in proximity to anoxic habitats containing methanogens. It was observed in the four lakes, but with a higher abundance in Lake Clément and Lake Saint-Charles (these lakes had, respectively, 65 and 32% of the reads associated with this genus). Although the surface waters of the studied lakes were almost fully oxygenated during the ice-cover period, the bottom waters had oxygen saturation values around 50%, with likely much lower oxygen tensions and possible anoxia in the lake sediments that would liberate reduced substrates into the water column. In contrast, the open-water period was characterized by the presence of cyanobacteria such as the bloom-forming *Microcystis* and *Dolichospermum*, as well as *Rabdoderma* and many unclassified picocyanobacteria.

Phytoplankton communities during the ice-cover period are generally dominated by either cyanobacteria or diatoms or by small heterotrophic or mixotrophic flagellates such as chrysophytes and cryptophytes (Öterler, 2017). The shift in dominance between these groups is mainly related to the trophic status of the lake, and the thickness of the snow and ice cover, with chrysophytes and cryptophytes negatively correlated with the former and positively with the latter (Beall et al., 2016; Kalinowska and Grabowska, 2016). During winter, the studied lakes were covered with 70 cm of ice and 40 cm of snow that would greatly attenuate the light, and lead to the expected dominance of chrysophytes and cryptophytes. These groups include mixotrophic taxa that have the competitive advantage of motility and of access to bacterial carbon and nutrients during periods of resource limitation (Bertilsson et al., 2013). However, while cryptophytes were almost exclusively found during the ice-cover period, chrysophytes also dominated during the open-water period, but not the same genera. There was also an increase in abundance of various chlorophytes during the ice-cover period, mainly small ovoid flagellates such as *Chlamydomonas* and *Oophila*. *Chlamydomonas* is frequently encountered during the ice-cover period, including in perennially ice covered systems (e.g., Bégin et al., 2020).

In the present study, the alpha-diversity (within sample) of prokaryotes was not influenced by season, but the alpha-diversity of eukaryotes was higher during open-water period with all methods. Along the same lines, there were more species/OTUs unique to the open-water period than to the ice-cover period, both for prokaryotes and microbial eukaryotes, as has been previously observed for prokaryotes (Wilhelm et al., 2014). While the ice-cover period offers niches that are not found during the open-water period, it is more stable in time and therefore limits species succession. The stability, however, leads to the establishment of physico-chemical gradients that may offer a variety of niches. In comparison, the open-water period goes through a low light mixing period with high nutrient levels (spring), a higher light stratified period with lower nutrient levels and bottom water hypoxia/anoxia (summer), a lower light mixing period with lower nutrients (autumn), and multiple transition stages. Furthermore, the greater connexion with the surrounding watershed during the open-water period, with stream and runoff inputs of allochthonous materials, would contribute to a greater diversity of available substrates.

### Comparison of Methods

One of our aims in this study was to compare a variety of methods to evaluate their complementarity and to provide a broad depiction of the taxonomic community structure. We were concerned that DNA analyses in winter could be biased toward dormant and degrading cells that were not active in the under-ice microbiome, and our molecular analyses therefore focused on RNA rather than DNA. Specifically, we targeted 16S or 18S rRNA (via conversion to cDNA) as a measure of ribosomal potential for protein synthesis and a more accurate guide to potential *in situ* activity than 16S or 18S rRNA genes (Blazewicz et al., 2013). While some studies have shown a correspondence between the two approaches (e.g., Mohit et al., 2017), there can be differences in regions or times of rapid successional change (e.g., Kalenitchenko et al., 2019).

The biggest differences in community composition observed between the methods was the over-representation of ciliates and the under-representation of diatoms with 18S rRNA, and the absence of dinoflagellates with chloroplast 16S rRNA. The disparity in the abundance of ciliates between 18S rRNA and microscopy has mainly been observed as a result of their high number of gene copies (Medinger et al., 2010). However, when the 18S rRNA (cDNA) is analyzed instead of 18S rDNA, there is a better correlation in the abundance of different groups between sequencing and microscopy (Giner et al., 2016; Pingping et al., 2020). In those studies, the size distribution of the compared groups was more uniform, because they were limited to picoeukaryotes or ciliates, respectively. In the present study, the bias toward ciliates might have arisen from size effects as the cellular quantity of rRNA increases with cell size (Fu and Gong, 2017), and ciliates are generally bigger than most of the other identified groups. It is also likely that delicate ciliates are differentially lost during the preservation of microscopy samples (Stoecker et al., 1994; Medinger et al., 2010). Lower diatom abundances in 18S rRNA reads have been attributed to the cell-lysis step of RNA/DNA extraction, which may be less efficient for this group due to their frustules (Medinger et al., 2010). The unusual attributes of dinoflagellate chloroplasts might explain why they were not amplified by the 16S rRNA primer when they were detected, sometimes in high relative abundance, with the three other methods; the genome of dinoflagellate chloroplasts is fragmented with certain gene functions transferred into the nucleus (Bennke et al., 2018). Furthermore, some members of this group are kleptoplastidic, with the ability to steal chloroplasts from other photosynthetic algae (Bennke et al., 2018). Some ciliates, such as the genus *Mesodinium* identified in the present study by 18S rRNA and microscopy, are also capable of kleptoplastidy (Bennke et al., 2018), which means that some of the chloroplast 16S rRNA reads might not represent the actual community, but the identity of the original bearers of the chloroplasts. Some of the differences might also arise from differences in transcription regulation between chloroplast and nuclear rRNA (Marín-Navarro et al., 2007). In interpreting these differences, the differential affinity and resolution of the primers for sequences of different groups and species at a particular location in their genome (e.g., V4) must also be considered (Rimet et al., 2018; Choi and Park, 2020). Compounds present in the initial water sample, such as humic materials in high concentration, can also interfere with nucleic acid extraction or inhibit the PCR steps (Crevecoeur et al., 2015).

Among all methods, 18S rRNA detected the highest α diversity (number of OTUs per sample), followed by microscopy and 16S rRNA in chloroplasts. The higher diversity using 18S rRNA as compared to microscopy has been attributed to its better detection of rare species due to higher total counts (in the present study there was around 6000 reads per sample with 18S rRNA, vs. 800 cells per sample by microscopy), its detection of cryptic or rare species, and to factors linked to sequencing errors and OTU groupings (Medinger et al., 2010; Rimet et al., 2018). The detection of species by microscopy is also limited by the skills of the analyst and the size of the cells (Karlson et al., 2010). Furthermore, some species are lost during the preservation protocol with acid Lugol’s (Gieskes and Kraay, 1983; Stoecker et al., 1994) or are altered by it, for example by break-up of colonies (Mukherjee et al., 2014), loss of flagella and discoloration (Karlson et al., 2010). Phase contrast and staining agents (e.g., silver staining of ciliates) can help with the identification of colorless or discolored species (Kim and Jung, 2017). The lowest diversity detected with chloroplast 16S rRNA was mainly due to the fact that it only included phototrophs. Furthermore, the database for chloroplast 16S rRNA is still limited (Bennke et al., 2018), and therefore many species are lumped together. The nature of certain chloroplasts, as mentioned for dinoflagellates, might also contribute to this lower resolution of species.

Microscopy, HPLC pigments and molecular analysis with either chloroplast 16S rRNA or 18S rRNA each provide useful ways to identify the factors driving differences in the taxonomic composition of microbial communities among sites, sampling times or experimental treatments. It is unusual to apply all five methods in a single study, but previous studies have used pairs of methods to differentiate communities: fluorescence (pigments) and microscopy (Lévesque et al., 2020); 18S rRNA/18S rRNA genes and microscopy (Gao et al., 2018; Rimet et al., 2018; Minerovic et al., 2020); and chloroplast 16S rRNA and microscopy (Eiler et al., 2013; Bennke et al., 2018). In the present study, 18S rRNA and chloroplast 16S rRNA offered better resolution for the separation of the samples among lakes and sample period than microscopy, as observed elsewhere (Yan et al., 2007). However, the combination of methods provides the best estimate of taxonomic composition of the microbial community, including by way of biovolume proportions that can be obtained by microscopy (Auinger et al., 2008; McManus and Katz, 2009; Xiao et al., 2014), albeit with the caveats noted above. These results also raise questions regarding the limitations of bacterial analysis, particularly for heterotrophic taxa that can only be identified by genomic approaches. These methods can additionally be augmented to consider functional activities and diversity by targeting specific genes using amplicons or transcriptomics to quantify ecologically relevant processes (e.g., methanotrophy; Crevecoeur et al., 2017), and by omics methods to functionally as well as taxonomically analyze the entire community (e.g., winter vs. summer microbiomes; Vigneron et al., 2019). Metagenomic analysis can also identify important bacterial groups that are poorly resolved or undetected by amplicon sequencing with standard primer sets (e.g., Candidate Phyla Radiation bacteria (Patescibacteria); Vigneron et al., 2020), but is still constrained by problems in sample handling and downstream bioinformatics analysis (Bharti and Grimm, 2021).

## Conclusion

Consistent with our hypothesis, there was a statistical relationship between community structure and chloride concentrations, notably on the relative abundance of cryptophytes and haptophytes, suggesting an influence of salinization across our sampled gradient of lakewater conditions. Large differences in trophic status and other limnological properties were insufficient to blanket out this effect, implying that the salt-rich runoff from roadside snowmelt may cause a shift in lake microbiomes during ice-cover as well as open-water periods. The application of diverse methods in the present study underscored the large differences between under-ice and open-water microbial communities. The seasonal differences included the tendency of the under-ice communities to have smaller algal cells, greater pigmentation, and higher relative abundances of methanotrophs, nitrifiers, and cryptophytes than the open-water communities. These results attest to the importance of extending limnological studies to all seasons, including winter, to capture the full range of microbiome compositions, functions and responses to contaminant exposure.

## Data Availability Statement

The datasets presented in this study can be found in online repositories. The names of the repository/repositories and accession number(s) can be found below: https://www.ncbi.nlm.nih.gov/, PRJNA681583.

## Author Contributions

IBF formulated the research question and designed the sampling, with input from WFV. IBF conducted the field sampling and the laboratory and data analyses. WFV provided funding. CL provided the infrastructure for the molecular analyses and advised IBF on the bioinformatics analyses. IBF led the writing of the manuscript with input from all authors.

## Conflict of Interest

The authors declare that the research was conducted in the absence of any commercial or financial relationships that could be construed as a potential conflict of interest.

## References

[B1] APEL (2011). *Association pour la protection de l’environnement du lac Saint-Charles et des Marais du Nord. Suivi du lac Clément Évaluation de la contamination par les sels de voirie.* Saint-Victor, QC: APEL.

[B2] APEL (2014). *Association pour la protection de l’environnement du lac Saint-Charles et des Marais du Nord. Diagnose du lac Saint-Charles, rapport final* Saint-Victor, QC: APEL.

[B3] APEL (2016). *Association pour la protection de l’environnement du lac Saint-Charles et des Marais du Nord. Lac Clair: Évaluation de l’état trophique, étude de la communauté cyanobactérienne, inventaire des herbiers aquatiques et évaluation de la conductivité spécifique - Faits saillants de la campagne d’échantillonnage 2015.* Saint-Victor, QC: APEL.

[B4] Apprill A. McNally S. Parsons R. Weber L. (2015). Minor revision to V4 region SSU rRNA 806R gene primer greatly increases detection of SAR11 bacterioplankton. *Aquat. Microb. Ecol.* 75 129–137. 10.3354/ame01753

[B5] Auinger B. M. Pfandl K. Boenigk J. (2008). Improved methodology for identification of protists and microalgae from plankton samples preserved in Lugol’s iodine solution: Combining microscopic analysis with single-cell PCR. *Appl. Environ. Microbiol.* 74 2505–2510. 10.1128/AEM.01803-07 18296536 PMC2293146

[B6] Balzano S. Abs E. Leterme S. C. (2015). Protist diversity along a salinity gradient in a coastal lagoon. *Aquat. Microb. Ecol.* 74 263–277. 10.3354/ame01740

[B7] Bashenkhaeva M. V. Zakharova Y. R. Petrova D. P. Khanaev I. V. Yury P. Likhoshway Y. V. (2015). Sub-ice microalgal and bacterial communities in freshwater Lake Baikal, Russia. *Microb. Ecol.* 70 751–765. 10.1007/s00248-015-0619-2 25933636

[B8] Bazin P. Jouenne F. Deton-Cabanillas A. F. Pérez-Ruzafa A. Véron B. (2014). Complex patterns in phytoplankton and microeukaryote diversity along the estuarine continuum. *Hydrobiologia* 726 155–178. 10.1007/s10750-013-1761-9

[B9] Beall B. F. N. Twiss M. R. Smith D. E. Oyserman B. O. Rozmarynowycz M. J. Binding C. E. (2016). Ice cover extent drives phytoplankton and bacterial community structure in a large north-temperate lake: implications for a warming climate: Effect of ice cover on microbial community structure. *Environ. Microbiol.* 18 1704–1719. 10.1111/1462-2920.12819 25712272

[B10] Bégin P. N. Rautio M. Tanabe Y. Uchida M. Culley A. I. Vincent W. F. (2020). The littoral zone of polar lakes: Inshore-offshore contrasts in an ice-covered High Arctic lake. *Arct. Sci.* 6:26. 10.1139/as-2020-0026

[B11] Bennke C. M. Pollehne F. Müller A. Hansen R. Kreikemeyer B. Labrenz M. (2018). The distribution of phytoplankton in the Baltic Sea assessed by a prokaryotic 16S rRNA gene primer system. *J. Plankton Res.* 40 244–254. 10.1093/plankt/fby008 32665766

[B12] Bertilsson S. Burgin A. Carey C. C. Fey S. B. Grossart H. P. Grubisic L. M. (2013). The under-ice microbiome of seasonally frozen lakes. *Limnol. Oceanogr.* 58 1998–2012. 10.4319/lo.2013.58.6.1998

[B13] Bharti R. Grimm D. G. (2021). Current challenges and best-practice protocols for microbiome analysis. *Brief. Bioinformat.* 22 178–193. 10.1093/bib/bbz155 31848574 PMC7820839

[B14] Bird D. L. Gro P. M. Salice C. J. Moore J. (2018). Steady-state land cover but non-steady-state major ion chemistry in urban streams. *Environ. Sci. Technol.* 52 13015–13026. 10.1021/acs.est.8b03587 30338996

[B15] Blazewicz S. J. Barnard R. L. Daly R. A. Firestone M. K. (2013). Evaluating rRNA as an indicator of microbial activity in environmental communities: limitations and uses. *ISME J.* 7 2061–2068. 10.1038/ismej.2013.102 23823491 PMC3806256

[B16] Bouvier T. C. del Giorgio P. A. (2002). Compositional changes in free-living bacterial communities along a salinity gradient in two temperate estuaries. *Limnol. Oceanogr.* 47 453–470. 10.4319/lo.2002.47.2.0453

[B17] Bushnell B. Rood J. Singer E. (2017). BBMerge – Accurate paired shotgun read merging via overlap. *PLoS One* 12:e0185056. 10.1371/journal.pone.0185056 29073143 PMC5657622

[B18] Butts E. Carrick H. J. (2017). Phytoplankton seasonality along a trophic gradient of temperate lakes: Convergence in taxonomic compostion during winter ice-cover. *Northeast Nat.* 24 B167–B187. 10.1656/045.024.s719 22708719

[B19] Casamayor E. O. Massana R. Benlloch S. Øvreås L. Díez B. Goddard V. J. (2002). Changes in archaeal, bacterial and eukaryal assemblages along a salinity gradient by comparison of genetic fingerprinting methods in a multipond solar saltern. *Environ. Microbiol.* 4 338–348. 10.1046/j.1462-2920.2002.00297.x 12071979

[B20] Cavaliere E. Baulch H. M. (2019). Winter nitrification in ice-covered lakes. *PLoS One* 14:e0224864. 10.1371/journal.pone.0224864 31697768 PMC6837456

[B21] CCME (2011). *Canadian Council of Ministers of the Environment. Scientific Criteria. Document for the development of the Canadian water quality guidelines for the protection of aquatic life: chloride ion.* Winnipeg, ON: CCME.

[B22] Celepli N. Sundh J. Ekman M. Dupont C. L. Yooseph S. Bergman B. (2017). Meta-omic analyses of Baltic Sea cyanobacteria: diversity, community structure and salt acclimation. *Environ. Microbiol.* 19 673–686. 10.1111/1462-2920.13592 27871145

[B23] Choi J. Park J. S. (2020). Comparative analyses of the V4 and V9 regions of 18S rDNA for the extant eukaryotic community using the Illumina platform. *Sci. Rep.* 10:6519. 10.1038/s41598-020-63561-z 32300168 PMC7162856

[B24] Comeau A. M. Li W. K. W. Tremblay É Carmack E. C. Lovejoy C. (2011). Arctic Ocean Microbial community structure before and after the 2007 record sea ice minimum. *PLoS One* 6:e27492. 10.1371/journal.pone.0027492 22096583 PMC3212577

[B25] Crevecoeur S. Vincent W. F. Comte J. Lovejoy C. (2015). Bacterial community structure across environmental gradients in permafrost thaw ponds: methanotroph-rich ecosystems. *Front. Microbiol.* 6:192. 10.3389/fmicb.2015.00192 25926816 PMC4396522

[B26] Crevecoeur S. Vincent W. F. Comte J. Matveev A. Lovejoy C. (2017). Diversity and potential activity of methanotrophs in high methane-emitting permafrost thaw ponds. *PLoS One* 12:e0188223. 10.1371/journal.pone.0188223 29182670 PMC5705078

[B27] Cruaud P. Vigneron A. Fradette M. S. Dorea C. C. Culley A. I. Rodriguez M. J. (2019b). Annual protist community dynamics in a freshwater ecosystem undergoing contrasted climatic conditions: The Saint-Charles River (Canada). *Front. Microbiol.* 10:2359. 10.3389/fmicb.2019.02359 31681222 PMC6805768

[B28] Cruaud P. Vigneron A. Fradette M. S. Dorea C. C. Culley A. I. Rodriguez M. J. (2019a). Annual bacterial community cycle in a seasonally ice-covered river reflects environmental and climatic conditions. *Limnol. Oceanogr.* 65 S21–S37. 10.1002/lno.11130

[B29] Dinno A. (2017). *dunn.test: Dunn’s Test of Multiple Comparisons Using Rank Sums. R package version 1.3.5.* Vienna: R Core Team.

[B30] Dos Santos V. M. de Andrade L. C. Tiecher T. de Oliveira Camargo F. A. (2020). The urban pressure over the sediment contamination in a southern Brazil metropolis: The case of Diluvio stream. *Water Air Soil Pollut.* 231:156. 10.1007/s11270-020-04504-2

[B31] Dugan H. A. Bartlett S. L. Burke S. M. Doubek J. P. Krivak-tetley F. E. Skaff N. K. (2017). Salting our freshwater lakes. *PNAS* 114 4453–4458. 10.1073/pnas.1620211114 28396392 PMC5410852

[B32] Edgar E. C. (2013). UPARSE: highly accurate OTU sequences from microbial amplicon reads. *Nat. Methods* 10 996–998. 10.1038/nmeth.2604 23955772

[B33] Eiler A. Drakare S. Bertilsson S. Pernthaler J. Peura S. Rofner C. (2013). Unveiling distribution patterns of freshwater phytoplankton by a Next Generation Sequencing based approach. *PLoS One* 8:e53516. 10.1371/journal.pone.0053516 23349714 PMC3551911

[B34] Eiler A. Zaremba-niedzwiedzka K. Martínez-garcía M. Mcmahon K. D. Stepanauskas R. Andersson S. G. E. (2014). Productivity and salinity structuring of the microplankton revealed by comparative freshwater metagenomics. *Environ. Microbiol.* 16 2682–2698. 10.1111/1462-2920.12301 24118837 PMC4253090

[B35] Fournier I. B. Galvez-Cloutier R. Vincent W. F. (2020). Roadside snowmelt: a management target to reduce lake and river contamination. *Inland Waters* 2020:1801312. 10.1080/20442041.2020.1801312

[B36] Fu R. Gong J. (2017). Single cell analysis linking ribosomal (r)DNA and rRNA copy numbers to cell size and growth rate provides insights into molecular protistan ecology. *J. Eukaryot. Microbiol.* 64 885–896. 10.1111/jeu.12425 28499076 PMC5697653

[B37] Gao W. Chen Z. Li Y. Pan Y. Zhu J. Guo S. (2018). Bioassessment of a drinking water reservoir using plankton: High Throughput Sequencing vs. traditional morphological method. *Water* 10:82. 10.3390/w10010082

[B38] Gieskes W. W. C. Kraay G. W. (1983). Dominance of Cryptophyceae during the phytoplankton spring bloom in the central North Sea detected by HPLC analysis of pigments. *Mar. Biol.* 75 179–185. 10.1007/BF00406000

[B39] Giner C. R. Forn I. Romac S. Logares R. de Vargas C. Massana R. (2016). Environmental sequencing provides reasonable estimates of the relative abundance of specific picoeukaryotes. *Appl. Environ. Microbiol.* 82 4757–4766. 10.1128/AEM.00560-16 27235440 PMC4984273

[B40] Government of Canada (2019). *Past weather and climate Database. 2019 – Historical data.* Canada: Government of Canada.

[B41] Greenwald G. M. Hurlbert S. H. (1993). Microcosm analysis of salinity effects on coastal lagoon plankton assemblages. *Hydrobiologia* 267 307–335. 10.1007/BF00018810

[B42] Grossart H. P. Massana R. McMahon K. D. Walsh D. A. (2020). Linking metagenomics to aquatic microbial ecology and biogeochemical cycles. *Limnol. Oceanogr.* 65 S2–S20. 10.1002/lno.11382

[B43] Gu Z. (2016). Complex heatmaps reveal patterns and correlations in multidimensional genomic data. *Bioinformatics* 32 2847–2849. 10.1093/bioinformatics/btw313 27207943

[B44] Guesdon G. de Santiago-Martin A. Raymond S. Messaoud H. Michaux A. Roy S. (2016). Impacts of salinity on Saint-Augustin Lake, Canada: Remediation measures at watershed scale. *Water* 8:285. 10.3390/w8070285

[B45] Guillou L. Bachar D. Audic S. Bass D. Berney C. Bittner L. (2013). The Protist Ribosomal Reference database (PR2): a catalog of unicellular eukaryote Small Sub-Unit rRNA sequences with curated taxonomy. *Nucleic Acids Res.* 41 D597–D604. 10.1093/nar/gks1160 23193267 PMC3531120

[B46] Herlemann D. P. R. Labrenz M. Jürgens K. Bertilsson S. Waniek J. J. Andersson A. F. (2011). Transitions in bacterial communities along the 2000 km salinity gradient of the Baltic Sea. *ISME J.* 5 1571–1579. 10.1038/ismej.2011.41 21472016 PMC3176514

[B47] Hillebrand H. Durselen C. D. Kirschtel D. Pollingher U. Zohary T. (1999). Biovolume calculation for pelagic and benthic microalgae. *J. Phycol.* 35 403–424. 10.1046/j.1529-8817.1999.3520403.x

[B48] Hoef-Emden K. (2014). Osmotolerance in the Cryptophyceae: Jacks-of-all-trades in the *Chroomonas* clade. *Protists* 165 123–143. 10.1016/j.protis.2014.01.001 24568876

[B49] Hong B. Lin Q. Yu S. Chen Y. Chen Y. Chiang P. (2018). Urbanization gradient of selected pharmaceuticals in surface water at a watershed scale. *Sci. Total Environ.* 634 448–458. 10.1016/j.scitotenv.2018.03.392 29631135

[B50] Houle D. Duchesne L. Ouimet R. Paquin R. Meng F. R. Arp P. A. (2002). Evaluation of the FORHYM2 model for prediction of hydrologic fluxes and soil temperature at the Lake Clair Watershed (Duchesnay, Quebec). *For. Ecol. Manag.* 159 249–260. 10.1016/S0378-1127(01)00438-8

[B51] Kalenitchenko D. Joli N. Potvin M. Tremblay J. É Lovejoy C. (2019). Biodiversity and species change in the Arctic Ocean: A view through the lens of Nares Strait. *Front. Mar. Sci.* 6:479. 10.3389/fmars.2019.00479

[B52] Kalinowska K. Grabowska M. (2016). Autotrophic and heterotrophic plankton under ice in a eutrophic temperate lake. *Hydrobiologia* 777 111–118. 10.1007/s10750-016-2769-8

[B53] Kalinowska K. Napiórkowska−Krzebietke A. Bogacka−Kapusta E. Stawecki K. (2019). Comparison of ice−on and ice−off abiotic and biotic parameters in three eutrophic lakes. *Ecol. Res.* 34 687–698. 10.1111/1440-1703.12039

[B54] Karlson B. Cusack C. Bresnan E. (eds) (2010). *Microscopic and molecular methods for quantitative phytoplankton analysis.* Paris: UNESCO.

[B55] Kaushal S. S. Likens G. E. Pace M. L. Utz R. M. Haq S. Gorman J. (2018). Freshwater salinization syndrome on a continental scale. *PNAS* 115 E574–E583. 10.1073/pnas.1711234115 29311318 PMC5789913

[B56] Kim J. H. Jung J. H. (2017). Cytological staining of protozoa: a case study on the impregnation of hypotrichs (Ciliophora: spirotrichea) using laboratory-synthesized protargol. *Anim. Cells Syst.* 21 412–418. 10.1080/19768354.2017.1376707

[B57] Legendre P. Anderson M. J. (1999). Distance-based redundancy analysis: Testing multispecies responses in multifactorial ecological experiments. *Ecol. Monogr.* 69:512. 10.1890/0012-96151999069[0001:DBRATM]2.0.CO;2

[B58] Lévesque D. Pinel-Alloul B. Giani A. Kufner D. C. L. Mimouni E. A. (2020). Are fluorometric, taxonomic, and functional indicators of phytoplankton community structure linked to environmental typology of urban ponds and lakes? *Inland Waters* 10 71–88. 10.1080/20442041.2019.1678970

[B59] Loewen C. J. G. Vinebrooke R. D. Zurawell R. W. (2021). Quantifying seasonal succession of phytoplankton trait−environment associations in human−altered landscapes. *Limnol. Oceanogr.* 2021:11694. 10.1002/lno.11694

[B60] Love M. I. Huber W. Anders S. (2014). Moderated estimation of fold change and dispersion for RNA-seq data with DESeq2. *Genome Biol.* 15:550. 10.1186/s13059-014-0550-8 25516281 PMC4302049

[B61] Mackey M. Mackey D. Higgins H. Wright S. (1996). CHEMTAX - a program for estimating class abundances from chemical markers: Application to HPLC measurements of phytoplankton. *Mar. Ecol. Prog. Ser.* 144 265–283. 10.3354/meps144265

[B62] Marín-Navarro J. Manuell A. L. Wu J. Mayfield S. P. (2007). Chloroplast translation regulation. *Photosynth. Res.* 94 359–374. 10.1007/s11120-007-9183-z 17661159

[B63] Martinez Arbizu P. (2020). *pairwiseAdonis: Pairwise multilevel comparison using adonis. R package version 0.0.1.* Vienna: R Core Team.

[B64] Massé S. Botrel M. Walsh D. A. Maranger R. (2019). Annual nitrification dynamics in a seasonally ice-covered lake. *PLoS One* 14:e0213748. 10.1371/journal.pone.0213748 30893339 PMC6426244

[B65] McManus G. B. Katz L. A. (2009). Molecular and morphological methods for identifying plankton: what makes a successful marriage? *J. Plankton Res.* 31 1119–1129. 10.1093/plankt/fbp061 32665766

[B66] Medinger R. Nolte V. Pandey R. V. Jost S. Ottenwälder B. Schlötterer C. (2010). Diversity in a hidden world: potential and limitation of next-generation sequencing for surveys of molecular diversity of eukaryotic microorganisms. *Mol. Ecol.* 19 32–40. 10.1111/j.1365-294X.2009.04478.x 20331768 PMC2953707

[B67] Minerovic A. D. Potapova M. G. Sales C. M. Price J. R. Enache M. D. (2020). 18S-V9 DNA metabarcoding detects the effect of water-quality impairment on stream biofilm eukaryotic assemblages. *Ecol. Indic.* 113:106225. 10.1016/j.ecolind.2020.106225

[B68] Mochizuki J. (2011). *Évaluation de la contamination du lac Clément, de son bassin versant et de la nappe phréatique par les sels de voirie – Charlesbourg, Québec.* Ph. D. thesis, Québec, QC: Université Laval.

[B69] Mohit V. Culley A. Lovejoy C. Bouchard F. Vincent W. F. (2017). Hidden biofilms in a far northern lake and implications for the changing Arctic. *NPJ Biofilms Microbiomes* 3:17. 10.1038/s41522-017-0024-3 28702216 PMC5500582

[B70] Moore J. Bird D. L. Dobbis S. K. Woodward G. (2017). Nonpoint source contributions drive elevated major ion and dissolved inorganic carbon concentrations in urban watersheds. *Environ. Sci. Technol. Lett.* 4 198–204. 10.1021/acs.estlett.7b00096

[B71] Mukherjee A. Das S. Bhattacharya T. De M. Maiti T. Kumar (2014). Optimization of phytoplankton preservative concentrations to reduce damage during long-term storage. *Biopreserv. Biobank.* 12 139–147. 10.1089/bio.2013.0074 24749881

[B72] Napiórkowska-Krzebietke A. Dunalska J. (2015). Phytoplankton-based recovery requirement for urban lakes in the implementation of the Water Framework Directive’s ecological targets. *Oceanol. Hydrobiol. Stud.* 44 109–119. 10.1515/ohs-2015-0011

[B73] Novotny E. V. Murphy D. Stefan H. G. (2008). Increase of urban lake salinity by road deicing salt. *Sci. Total Environ.* 406 131–144. 10.1016/j.scitotenv.2008.07.037 18762321

[B74] O’brien J. E. Majewaki J. C. (1975). Effects of de-icing salt on ground water characteristics. *Environ. Lett.* 8 303–313. 10.1080/00139307509437440 238830

[B75] OBV de la Capitale (2018). *Organisme des Bassins Versants de la Capitale. Diagnose du lac Saint-Augustin - Campagnes de Terrain 2014-2015. Pour la Ville de Saint-Augustin-de-Desmaures.* Québec, QC: OBV de la Capitale.

[B76] Oksanen J. Blanchet F. G. Friendly M. Kindt R. Legendre P. McGlinn D. (2019). *vegan: Community Ecology Package. R package version 2.5-6.* Vienna: R Core Team.

[B77] Ondarza P. M. Haddad S. P. Avigliano E. Miglioranza K. S. B. Brooks B. W. (2019). Pharmaceuticals, illicit drugs and their metabolites in fish from Argentina: Implications for protected areas influenced by urbanization. *Sci. Tot. Environ.* 649 1029–1037. 10.1016/j.scitotenv.2018.08.383 30308876

[B78] Öterler B. (2017). Winter phytoplankton composition occurring in a temporarily ice-covered lake: a case study. *Pol. J. Environ. Stud.* 26 2677–2688. 10.15244/pjoes/74015

[B79] Pingping H. Feng Z. Kuidong X. (2020). Complementary DNA sequencing (cDNA): an effective approach for assessing the diversity and distribution of marine benthic ciliates along hydrographic gradients. *Limnol. Oceanogr.* 2020:2. 10.1007/s00343-020-9234-2

[B80] Quast C. Pruesse E. Yilmaz P. Gerken J. Schweer T. Yarza P. (2013). The SILVA ribosomal RNA gene database project: improved data processing and web-based tools. *Nucleic Acids Res.* 41 D590–D596. 10.1093/nar/gks1219 23193283 PMC3531112

[B81] R Core Team (2013). *R: A language and environment for statistical computing.* Vienna: R Foundation for Statistical Computing.

[B82] Rimet F. Vasselon V. Keszte B. A. Bouchez A. (2018). Do we similarly assess diversity with microscopy and high-throughput sequencing? Case of microalgae in lakes. *Org. Divers. Evol.* 18 51–62. 10.1007/s13127-018-0359-5

[B83] Roberge K. (2004). *Paléolimnologie du Lac Saint-Augustin. Reconstitution de l’histoire trophique par 1’etude des diatomées fossiles, des pigments d’algues et de la géochimie des sédiments.* Ph. D. thesis, Québec, QC: Université Laval.

[B84] Rognes T. Flouri T. Nichols B. Quince C. Mahé F. (2016). VSEARCH: a versatile open source tool for metagenomics. *PeerJ* 4:e2584. 10.7717/peerj.2584 27781170 PMC5075697

[B85] Roy S. Llewellyn C. A. Skarstad E. Johnsen G. (2011). *Phytoplankton Pigments Characterization Chemotaxonomy and Applications in Oceanography.* Cambridge: Cambridge University Press.

[B86] Sakai R. Winand R. Verbeiren T. Vande Moere A. Aerts J. (2015). dendsort: modular leaf ordering methods for dendrogram representations in R [version 1; peer review: 2 approved]. *F1000Research* 3:177. 10.12688/f1000research.4784.1 25232468 PMC4162509

[B87] Saoud P. Kreydiyyeh S. Chalfoun A. Fakih M. (2007). Influence of salinity on survival, growth, plasma osmolality and gill Na^+^-K^+^-ATPase activity in the rabbitfish *Siganus rivulatus*. *J. Exp. Mar. Biol. Ecol.* 348 183–190. 10.1016/j.jembe.2007.05.005

[B88] Schloss P. D. Westcott S. L. Ryabin T. Hall J. R. Hartmann M. Hollister E. B. (2009). Introducing mothur: open-source, platform-independent, community-supported software for describing and comparing microbial communities. *Appl. Environ. Microbiol.* 75 7537–7541. 10.1128/AEM.01541-09 19801464 PMC2786419

[B89] Schluter L. Lauridsen T. L. Krogh G. Jorgensen T. (2006). Identification and quantification of phytoplankton groups in lakes using new pigment ratios – a comparison between pigment analysis by HPLC and microscopy. *Freshw. Biol.* 51 1474–1485. 10.1111/j.1365-2427.2006.01582.x

[B90] Scott R. Goulden T. Letman M. Hayward J. Jamieson R. (2019). Long-term evaluation of the impact of urbanization on chloride levels in lakes in a temperate region. *J. Environ. Manage.* 244 285–293. 10.1016/j.jenvman.2019.05.029 31128333

[B91] Shanley J. B. (1994). Effects of ion-exchange on stream solute fluxes in a basin receiving highway deicing salts. *J. Environ. Qual.* 23 977–986. 10.2134/jeq1994.00472425002300050019x 34872214

[B92] Stoecker D. Gifford D. Putt M. (1994). Preservation of marine planktonic ciliates: losses and cell shrinkage during fixation. *Mar. Ecol. Prog. Ser.* 110 293–299. 10.3354/meps110293

[B93] Tian Z. Zhao H. Peter K. T. Gonzalez M. Wetzel J. Wu C. (2020). A ubiquitous tire rubber–derived chemical induces acute mortality in Coho salmon. *Science* 371 185–189. 10.1126/science.abd6951 33273063

[B94] Toporowska M. Ferencz B. Dawidek J. (2018). Impact of lake-catchment processes on phytoplankton community structure in temperate shallow lakes: Impact of lake-catchment processes on phytoplankton. *Ecohydrology* 11:e2017. 10.1002/eco.2017

[B95] Van den Meersche K. Soetaert K. Middelburg J. J. (2008). A Bayesian compositional estimator for microbial taxonomy based on biomarkers: Bayesian compositional estimator. *Limnol. Oceanogr. Meth.* 6 190–199. 10.4319/lom.2008.6.190

[B96] Van Meter R. J. Swan C. M. Leips J. Snodgrass J. W. (2011). Road salt stress induces novel food web structure and interactions. *Wetlands* 31 843–851. 10.1007/s13157-011-0199-y

[B97] Vigneron A. Cruaud P. Langlois V. Lovejoy C. Culley A. I. Vincent W. F. (2020). Ultra-small and abundant: Candidate Phyla Radiation bacteria are potential catalysts of carbon transformation in a thermokarst lake ecosystem. *Limnol. Oceanogr. Lett.* 5 212–220. 10.1002/lol2.10132

[B98] Vigneron A. Lovejoy C. Cruaud P. Kalenitchenko D. Culley A. Vincent W. F. (2019). Contrasting winter versus summer microbial communities and metabolic functions in a permafrost thaw lake. *Front. Microbiol.* 10:1656. 10.3389/fmicb.2019.01656 31379798 PMC6646835

[B99] Wallace A. M. Biastoch R. G. (2016). Detecting changes in the benthic invertebrate community in response to increasing chloride in streams in Toronto, *Canada*. *Freshw. Sci.* 35 353–363. 10.1086/685297

[B100] Watanabe S. Laurion I. Chokmani K. Pienitz R. Vincent W. F. (2011). Optical diversity of thaw ponds in discontinuous permafrost: A model system for water color analysis. *J. Geophys. Res.* 116:G02203. 10.1029/2010JG001380

[B101] Wegen S. Nowka B. Spieck E. (2019). Low temperature and neutral pH define “Candidatus *Nitrotoga* sp.” as a competitive nitrite oxidizer in coculture with *Nitrospira defluvii*. *Appl. Environ. Microbiol.* 85 2569–2518e. 10.1128/AEM.02569-18 30824434 PMC6495747

[B102] Wetzel R. B. Likens G. (2000). *Limnological analyses*, 3rd Edn. New York: Springer.

[B103] Wilhelm S. W. LeCleir G. R. Bullerjahn G. S. McKay R. M. Saxton M. A. Twiss M. R. (2014). Seasonal changes in microbial community structure and activity imply winter production is linked to summer hypoxia in a large lake. *FEMS Microbiol. Ecol.* 87 475–485. 10.1111/1574-6941.12238 24164471

[B104] Xiao X. Sogge H. Lagesen K. Tooming-Klunderud A. Jakobsen K. S. Rohrlack T. (2014). Use of High Throughput Sequencing and light microscopy show contrasting results in a study of phytoplankton occurrence in a freshwater environment. *PLoS One* 9:e106510. 10.1371/journal.pone.0106510 25171164 PMC4149573

[B105] Yan Q. Y. Yu Y. H. Feng W. S. Deng W. N. Song X. H. (2007). Genetic diversity of plankton community as depicted by PCR-DGGE fingerprinting and its relation to morphological composition and environmental factors in Lake Donghu. *Microb. Ecol.* 54 290–297. 10.1007/s00248-006-9200-3 17541768

[B106] Zapata M. Jeffrey S. W. Wright S. W. Rodríguez F. Garrido J. L. Clementson L. (2004). Photosynthetic pigments in 37 species (65 strains) of Haptophyta: implications for oceanography and chemotaxonomy. *Mar. Ecol. Prog. Ser.* 270 83–102. 10.3354/meps270083

