# Supplementary material for: Changes in the Community Structure of Under-Ice and Open-Water Microbiomes in Urban Lakes Exposed to Road Salts
Source: Front Microbiol. 2021 Mar 31;12:660719. doi: 10.3389/fmicb.2021.660719 (PMC8044900; doi:10.3389/fmicb.2021.660719)
Supplement: Supplementary file 1 [file Data_Sheet_1.pdf]

Supplementary Material

Changes in the Community Structure of Under-Ice and Open-Water Microbiomes in Urban Lakes Exposed to Road Salts

Isabelle B. Fournier, Connie Lovejoy, Warwick F. Vincent

Supplementary Figures and Tables

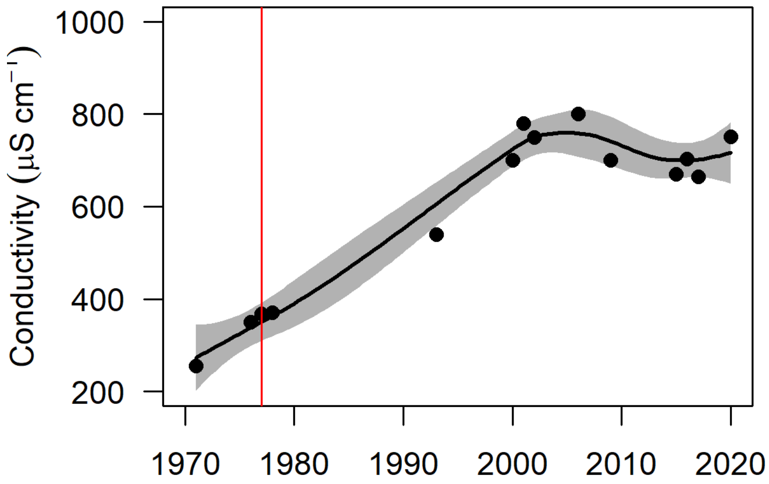

Supplementary Figure 1. Changes in the specific conductivity of Lake Saint-Augustin surface water from 1971 to present. Red line marks the Highway 40 construction in the lake watershed (1977, Pienitz et al. 2006). Black line is a degree 2 polynomial fit of span 0.75.

Sources: 1976-1978 Meunier and Alain 1979; 1993-2009 Galvez-Cloutier et al. 2012 and reference therein; 2015 OBV de la Capitale 2018; 2016-2017 present study; 13 September 2020, RBR-620-CTD, W.F. Vincent.

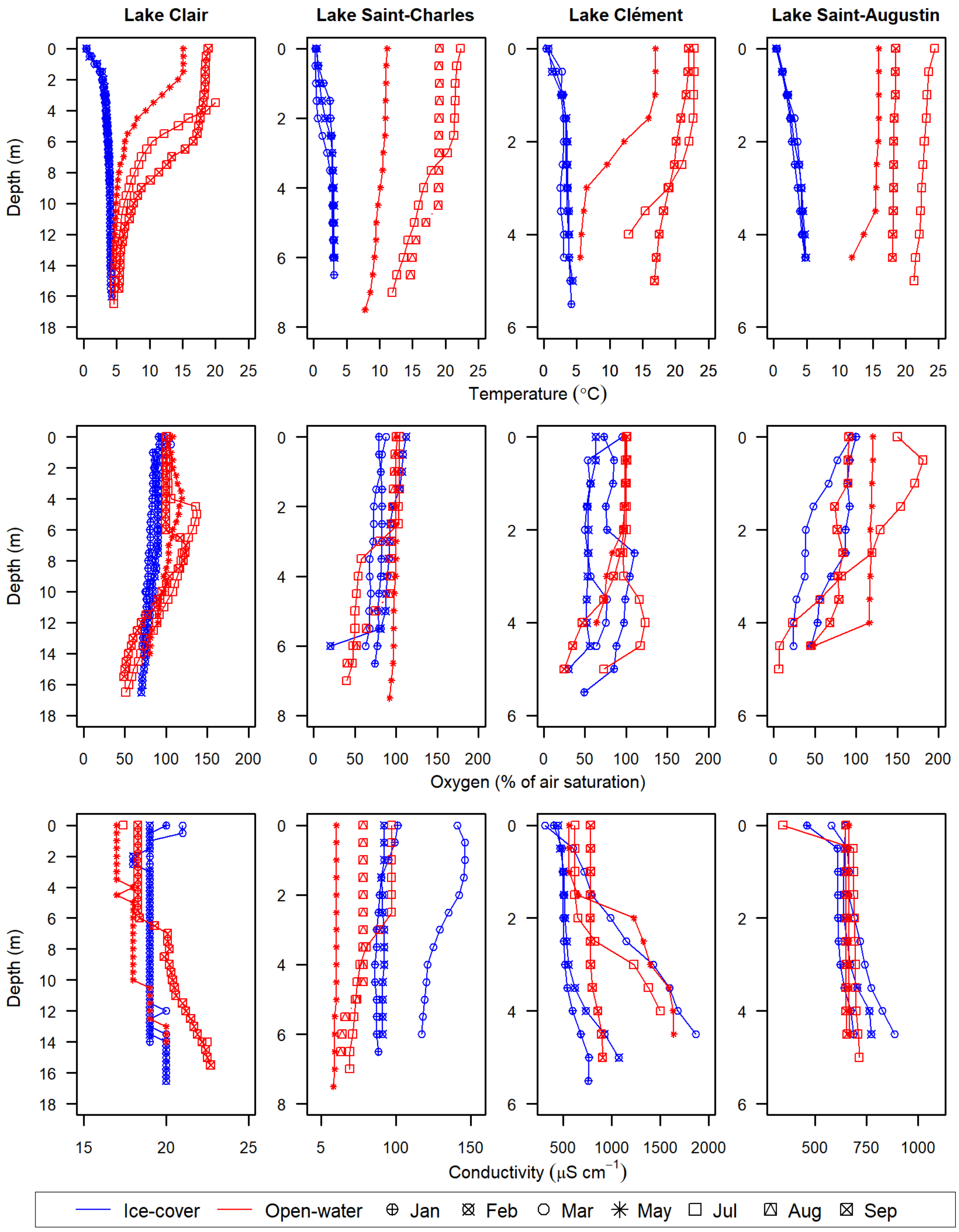

Supplementary Figure 2. Temperature, oxygen and conductivity profiles of Lake Clair, Lake Saint-Charles, Lake Clément and Lake Saint-Augustin in 2017.

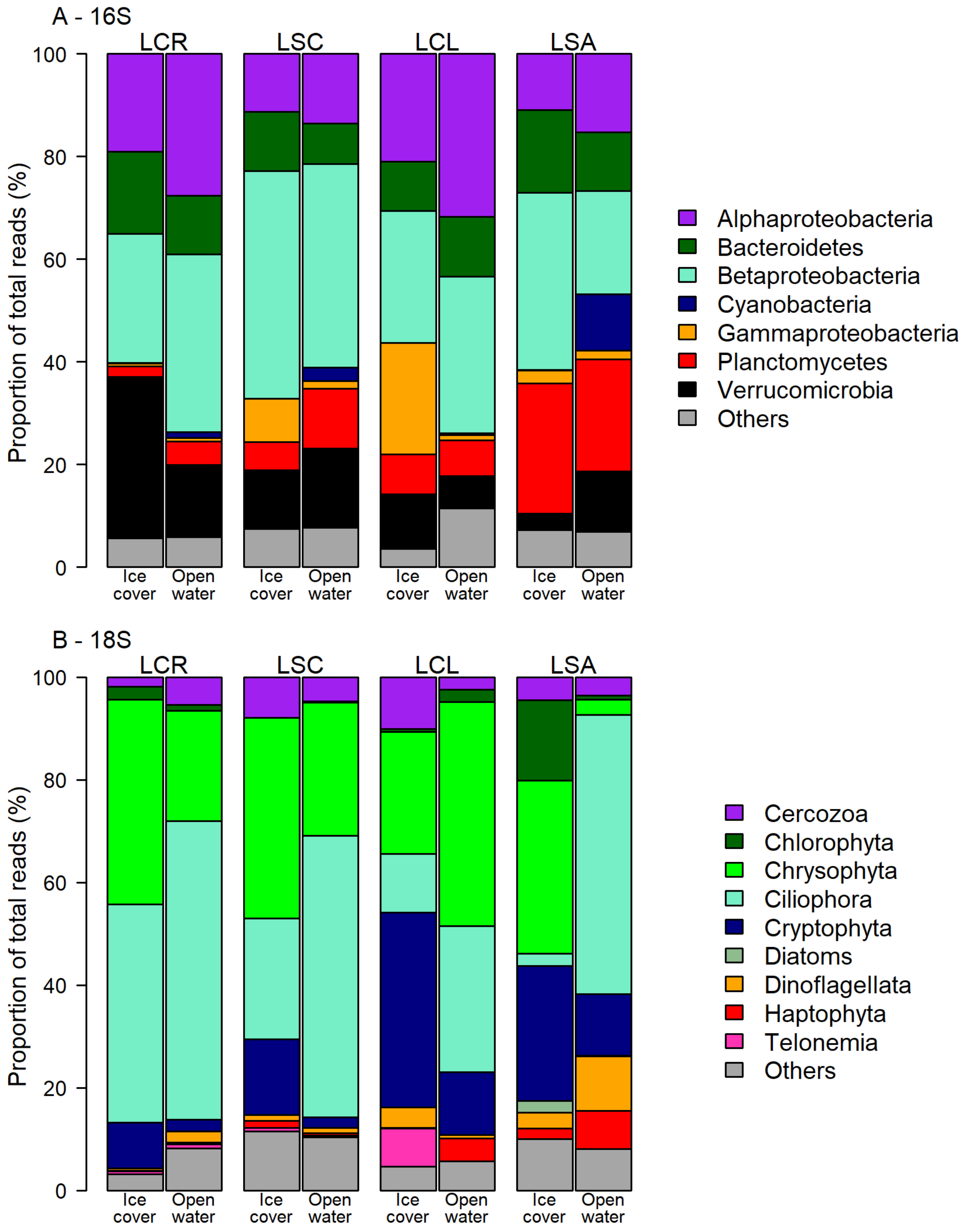

Supplementary Figure 3. Taxonomic composition at the phylum level for **(A)** Bacteria and **(B)** microbial eukaryotes from the rRNA analysis for Lake Clair, Lake Saint-Charles, Lake Clément, and Lake Saint-Augustin during the ice-cover and the open-water periods.

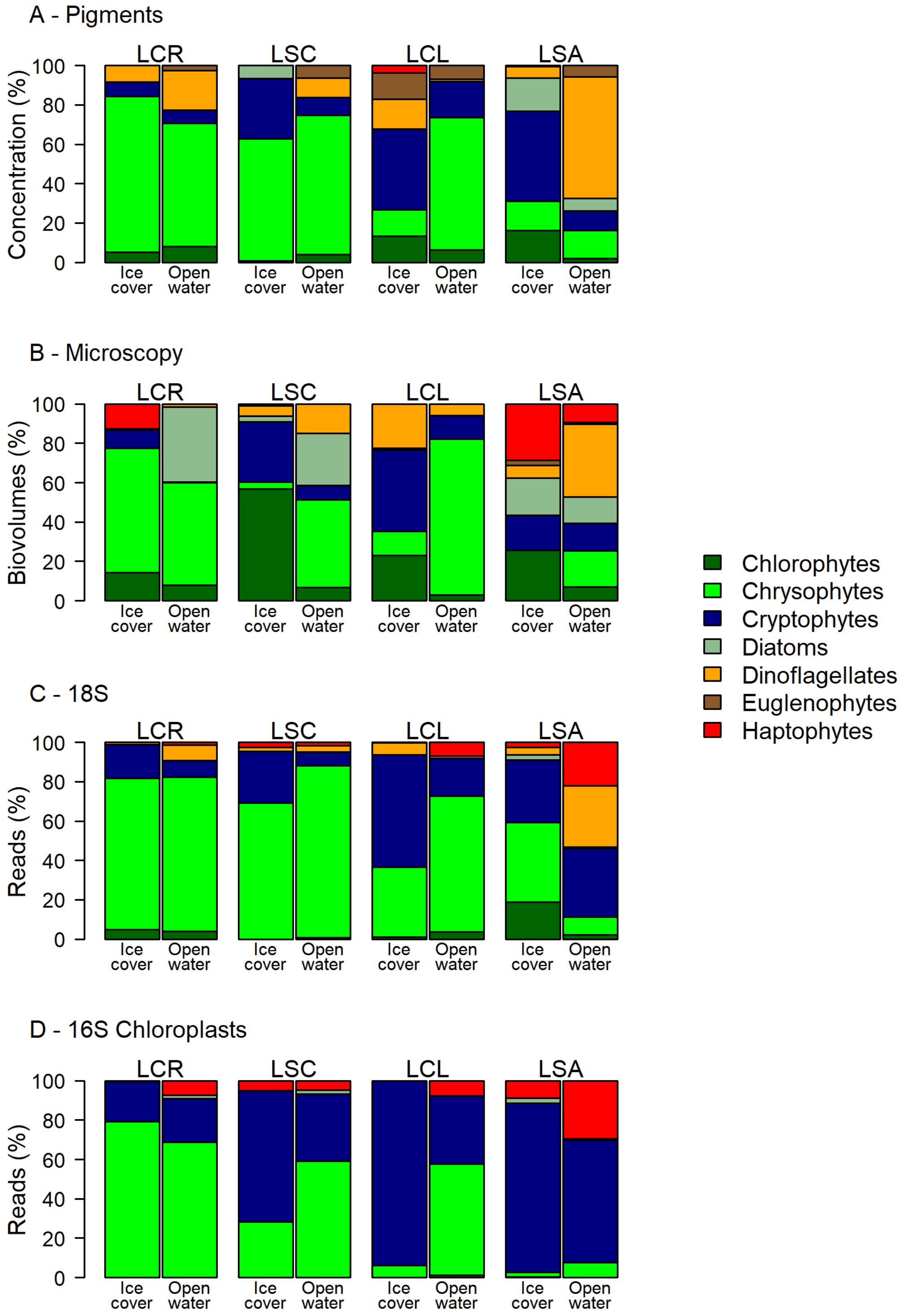

Supplementary Figure 4. Taxonomic composition at the phylum level for phytoplankton from **(A)** photosynthetic pigments, **(B)** microscopy, **(C)** 18S rRNA and **(D)** 16S rRNA chloroplasts for Lake Clair, Lake Saint-Charles, Lake Clément, and Lake Saint-Augustin during the ice-cover and the open-water periods.

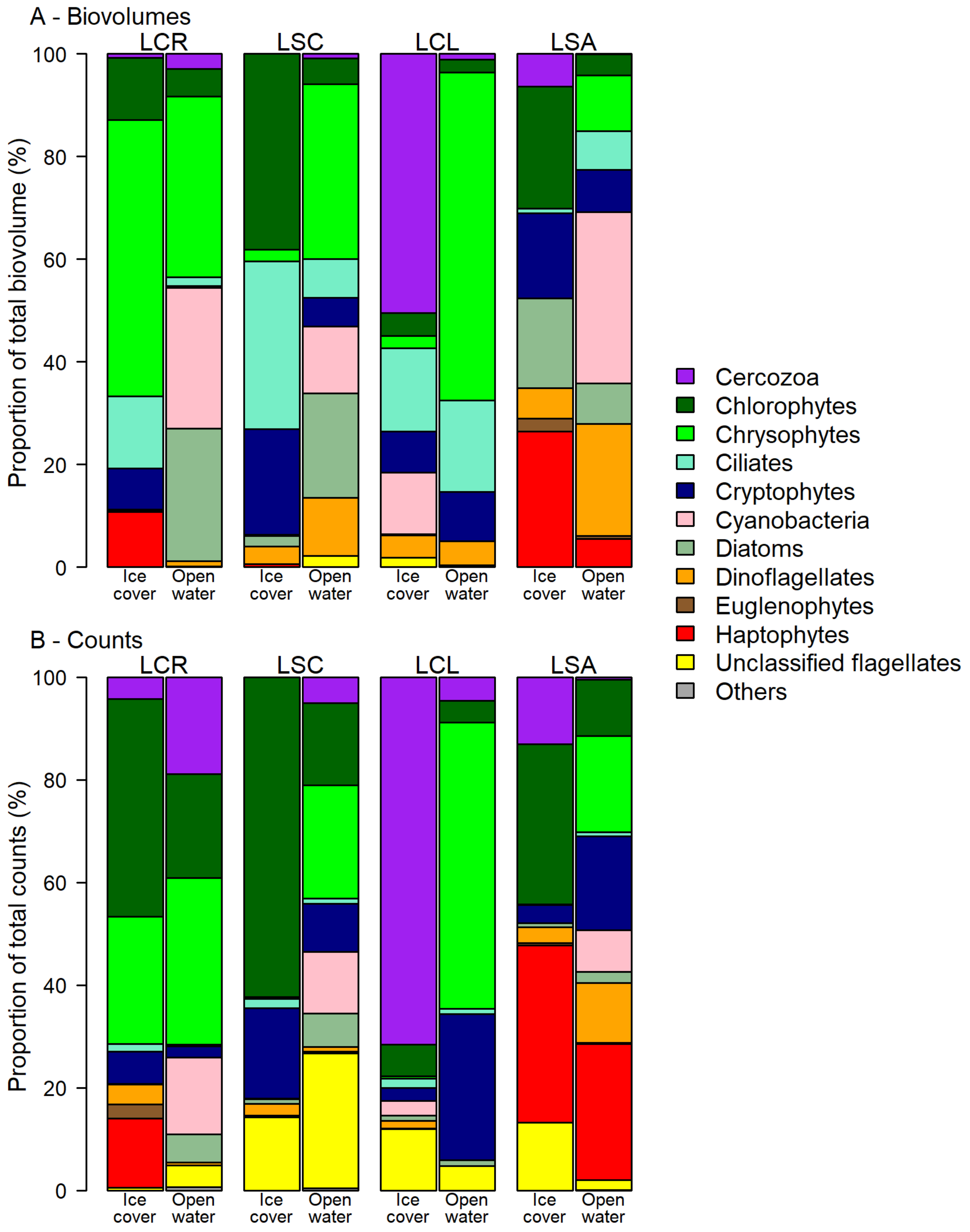

Supplementary Figure 5. Taxonomic composition at the phylum level for **(A)** Biovolumes and **(B)** counts derived from microscopy for Lake Clair, Lake Saint-Charles, Lake Clément, and Lake Saint-Augustin during the ice-cover and the open-water periods.

Supplementary Table 1. Sampling dates for Lake Clair, Lake Saint-Charles, Lake Clément, and Lake Saint-Augustin in 2017.

Season	Lake Clair	Lake Saint-Charles	Lake Clément	Lake Saint-Augustin
Winter	January 24	January 23	January 10	January 12
	February 21	February 23	February 9	February 7
	March 21	March 23	March 9	March 7
Spring	May 30	May 19	June 01	May 22
Summer	July 24	July 25	July 13	July 14
Autumn	September 18	August 24	September 26	September 13
		August 31		

Supplementary Table 2. Photosynthetic pigment mass ratios to chlorophyll *a* for different groups of photosynthetic plankton, used as input to the HPLC analysis. Carotene is the sum of β,α-carotene and β,β-carotene, and Chl *c* is the sum of chlorophyll *c1* and chlorophyll *c2*.

Pigments	Chlorophytes	Chrysophytes	Cryptophytes	Cyanobacteria	Diatoms	Dinoflagellates	Euglenophytes	Haptophytes
Alloxanthin	0	0	0.368	0	0	0	0	0
Antheraxanthin	0.016	0	0	0	0	0	0	0
Aphanizophyll	0	0	0	0.0054	0	0	0	0
Astaxanthin	0.004	0	0	0	0	0	0	0
Canthaxanthin	0	0	0	0	0	0	0.014	0
Carotene	0.003	0.003	0.004	0.097	0.003	0.0025	0.004	0.03
Chl *b*	0.356	0	0	0	0	0	0.198	0
Chl *c*	0	0.032	0.091	0	0.091	0.1555	0	0
Diadinoxanthin	0	0.016	0	0	0.064	0.2465	0.327	0.165
Diatoxanthin	0	0.025	0	0	0.083	0.0795	0	0.235
Dinoxanthin	0	0	0	0	0	0.053	0	0
Echinenone	0	0	0	0.526	0	0	0.026	0
Fucoxanthin	0	0.283	0	0	0.458	0	0	0.637
Lutein	0.147	0	0	0	0	0	0	0
Myxoxanthophyll	0	0	0	0.136	0	0	0	0
Neoxanthin	0.04	0	0	0	0	0	0.034	0
Peridinin	0	0	0	0	0	0.5075	0	0
Violaxanthin	0.026	0.063	0	0	0.003	0	0	0
Zeaxanthin	0.036	0.016	0	0.28	0.007	0	0	0

Supplementary Table 3. Conditions of the polymerase chain reaction (PCR) thermal cycling for the 515F/806R primers as modified by Aprill et al. (2015) and the 572F/1009R primers from Comeau et al. (2011).

**Steps**	**515F/806R**			**1389F/1510R**		
	Temperature (°C)	Time (seconds)	Cycles	Temperature (°C)	Time (seconds)	Cycles
Initial denaturation	None		NA	98	30	1
Denaturation	94	45	36	98	10	30
Annealing	50	60		52	30	
Extension	72	90		72	30	
Final extension	72	60	1	72	270	1

Supplementary Table 4. Most abundant taxa (OTUs were pooled at their lowest taxonomic ranks) in Lake Clair for 16S rRNA, 18S rRNA, microscopy, and chloroplast 16S rRNA during the open-water period; %reads: mean relative abundance in % of total reads, Biovolume%: mean relative abundance in % of total biovolume, CV%: coefficient of variation, SD as % mean.

Lake Clair open water

16S rRNA

Taxonomy	Reads%	CV%
Unclassified	28.5	10
Unclassified	20.5	98
Unclassified	11.0	99
Polynucleobacter sp.	3.5	34
Rhodovarius sp.	3.5	69
CL500-3	3.4	102
Sediminibacterium sp.	1.9	77
Actinobacteria unclassified	1.8	38
Flavobacterium sp.	1.6	135
Unclassified	1.5	96
Emticicia sp.	1.5	118
GKS98	1.3	10
Actinobacteria hgcI clade	1.2	45
Dinghuibacter sp.	1.2	102
Caulobacter sp.	0.9	62
Ferruginibacter sp.	0.9	75
Arcicella sp.	0.7	172
IMCC26134	0.7	167
SM1A02	0.7	155
Roseomonas sp.	0.6	94

18S rRNA

Taxonomy	Reads%	CV%
Mesodinium sp.	44.1	52
Chrysophyceae	7.7	75
Unclassified	4.4	146
Litostomatea	4.3	82
StrombidiidaA	3.8	97
Chrysophyceae cladeD	3.5	69
Chrysophyceae cladeC	2.4	63
Dinobryon sp.	2.4	35
Unclassified ochrophyta	2.1	110
Pterocystida	2.0	171
Unclassified	1.7	86
Cryptomonas pyrenoidifera	1.7	81
Unclassified	1.5	76
Chrysosphaerella sp.	1.2	149
Dinobryon crenulatum	0.9	67
Chrysophyceae cladeE	0.9	39
Askenasia sp.	0.8	56
Mamiellophyceae	0.8	118
Centroheliozoa	0.8	157
StrobilidiidaeC	0.7	114

Microscopy

Taxonomy	Biovolume%	CV%
Dinobryon bavaricum	29.1	69
Cyanobacteria unclassified	20.7	169
Chrysochromulina sp.	15.9	173
Picocyanobacteria	12.1	120
Chlamydomonas sp.	5.9	106
Unclassified	4.2	136
Dinobryon divergens	2.9	173
Strobilidium sp.	1.8	173
Peridinium sp.1	1.4	146
Oocystis sp.	1.1	173
Tabellaria fenestrata	0.8	173
Uronema sp.	0.5	173
Cryptomonas marssonii	0.5	104
Rabdoderma sp.	0.5	110
Merismopedia sp.	0.3	173
Unclassified flagellate	0.2	173
Staurastrum sp.	0.2	173
Peridinium sp.2	0.2	173
Urosolenia sp.	0.1	173
Mallomonas tonsurata	0.1	87

Chloroplast 16S rRNA

Taxonomy	Reads%	CV%
Unclassified	18.3	95
Synura uvella	13.8	118
Cryptomonas curvata	12.3	77
Florenciella parvula	11.7	70
Dinobryon LO226KS	7.9	59
Epipyxis PR26KG	7.6	56
Chrysochromulina CCMP291	4.1	116
Chrysosphaera sp.	2.8	145
Cryptomonas erosa	2.6	47
Ochromonas CCMP1393	2.0	45
Rhodophytes unclassified	1.7	53
Hemiselmis sp.	1.5	172
Acanthoceras zachariasii	1.3	161
Unclassified	1.0	87
Unclassified	0.6	55
Mallomonas splendens	0.5	126
Chromulina sp.	0.4	85
Pavlova gyrans	0.3	105
Pseudopedinella elastica	0.3	73
Oophila amblystomatis	0.1	87

Legend 16S rRNA

- Alphaproteobacteria
- Bacteroidetes
- Betaproteobacteria
- Cyanobacteria
- Gammaproteobacteria
- Planctomycetes
- Verrucomicrobia
- Others

Legend 18S rRNA, microscopy and chloroplast 16S rRNA

- Cercozoa
- Chlorophyta
- Ciliophora
- Cryptophyta
- Dinoflagellata
- Haptophyta
- Katablepharidophyta
- Telonemia
- Ochrophyta
- Others
- Unknown

Supplementary Table 5. Most abundant taxa (OTUs were pooled at their lowest taxonomic ranks) in Lake Clair for 16S rRNA, 18S rRNA, microscopy, and chloroplast 16S rRNA during the ice-cover period (Jan-Feb-Mar); %reads: mean relative abundance in % of total reads, Biovolume%: mean relative abundance in % of total biovolume, CV%: coefficient of variation, SD as % mean.

Lake Clair ice-cover

16S rRNA

Taxonomy	Reads %	CV%
Unclassified	21.1	13
Unclassified	15.5	3
Unclassified	13.1	30
Chthoniobacter sp.	6.4	37
Sediminibacterium sp.	5.4	71
SH3-11	3.9	60
Unclassified	3.2	46
BSV13	2.6	86
Luteolibacter sp.	2.5	152
Cephaloticoccus sp.	1.9	74
Flavobacterium sp.	1.8	21
Unclassified	1.8	65
Actinobacteria unclassified	1.6	44
Polynucleobacter sp.	1.6	48
Actinobacteria hgcI clade	1.5	33
Caulobacter sp.	1.0	96
Flexibacteraceae	0.9	87
Rhodovastum sp.	0.9	57
Marine group	0.8	119
Polaromonas sp.	0.8	57

18S rRNA

Taxonomy	Reads %	CV%
Mesodinium sp.	39.8	60
Synura mollispina	35.8	72
Unclassified cryptophyceae	5.2	90
Hamakko caudatus	2.4	69
Cryptomonas pyrenoidifera	2.9	81
Novel clade 10	1.2	28
Cryptomonas curvata	1.1	68
StrombidiidaA	0.9	139
Cryptomonadales	0.8	131
Katablepharidales	0.8	64
Ochrophyta	0.8	85
Chrysophyceae	0.7	87
Mallomonas sp.	0.6	78
Group 2	0.6	94
Pseudodendromonadales	0.6	80
Chrysophyceae cladeD	0.5	110
Cryptomonas tetrapyrenoidosa	0.5	101
Unclassified	0.4	121
Unclassified	0.4	134
Choanoflagellatea	0.4	155

Microscopy

Taxonomy	Biovolume %	CV%
Dinobryon divergens	46.0	132
Strobilidium sp.	12.0	173
Chlamydomonas sp.	11.2	84
Chrysochromulina sp.	10.8	173
Cryptomonas ovata	7.9	120
Dinobryon bavaricum	5.1	145
Mallomonas caudata	2.8	173
Uronema sp.	1.7	93
Unclassified	0.8	173
Cryptomonas marssonii	0.6	107
Unclassified flagellate 2	0.5	112
Mesodinium sp.	0.4	173
Chloromonas sp.	0.4	173
Peridinium sp.1	0.3	173
Eutreptia sp.	0.1	100
Unclassified	0.1	173
Cymbella sp.	0.0	173

Chloroplast 16S rRNA

Taxonomy	Reads %	CV%
Synura uvella	68.6	81
Cryptomonas erosa	10.6	37
Florenciella parvula	5.4	96
Cryptomonas curvata	4.6	48
Unclassified	4.1	63
Unclassified	3.0	127
Teleaulax amphioxeia	1.0	130
Epipyxis PR26KG	0.8	120
Unclassified	0.4	49
Dinobryon LO226KS	0.4	68
Heterosigma akashimo	0.4	59
Chrysochromulina CCMP291	0.1	147
Rhodophytes unclassified	0.1	124
Pavlova gyrans	0.1	87
Chrysosphaera sp.	0.1	136
Ochromonas CCMP1393	0.1	173
Cryptomonas ovata	0.1	87
Chromulina sp.	0.1	173
Mallomonas splendens	0.0	173
Unclassified	0.0	173

Legend 16S rRNA

- Alphaproteobacteria
- Bacteroidetes
- Betaproteobacteria
- Cyanobacteria
- Gammaproteobacteria
- Planctomycetes
- Verrucomicrobia
- Others

Legend 18S rRNA, microscopy and chloroplast 16S rRNA

- Cercozoa
- Chlorophyta
- Ciliophora
- Cryptophyta
- Dinoflagellata
- Haptophyta
- Katablepharidophyta
- Telonemia
- Ochrophyta
- Others
- Unknown

Supplementary Table 6. Most abundant taxa (OTUs were pooled at their lowest taxonomic ranks) in Lake Saint-Charles for 16S rRNA, 18S rRNA, microscopy, and chloroplast 16S rRNA during the open-water period; %reads: mean relative abundance in % of total reads, Biovolume%: mean relative abundance in % of total biovolume, CV%: coefficient of variation, SD as % mean.

Lake Saint-Charles open water

16S rRNA			18S rRNA			Microscopy			Chloroplast 16S rRNA		
Taxonomy	Reads %	CV%	Taxonomy	Reads %	CV%	Taxonomy	Biovolume %	CV%	Taxonomy	Reads %	CV%
Unclassified	31.1	104	Litostomatea	28.6	103	*Synura uvella*	17.2	148	*Florenciella parvula*	18.6	122
Unclassified	11.2	40	*Mesodinium* sp.	6.2	124	Picocyanobacteria	12.1	72	Unclassified	15.7	65
Phycispaerae CL500-3	10.7	85	*Dinobryon sociale*	3.6	72	*Chrysochromulina* sp.	10.2	136	*Cryptomonas erosa*	11.1	32
Unclassified	8.9	63	*Pseudoholophrya* sp.	3.5	135	*Ceratium hirundinella*	9.3	200	*Cryptomonas curvata*	8.3	60
Polynucleobacter sp.	3.8	132	Unclassified chrysophyceae	3.2	51	*Asterionella formosa*	7.7	130	*Epipyxis* PR26KG	7.8	55
Sediminibacterium sp.	2.5	185	*Dinobryon divergens*	3.1	122	*Dinobryon bavaricum*	6.9	70	*Synura uvella*	7.1	56
Actinobacteria unclassified	2.3	154	Chrysophyceae cladeD	2.8	84	*Strobilidium* sp.	6.6	71	*Dinobryon* LO226KS	6.6	46
Unclassified	1.7	80	StrombidiidaA	2.7	106	*Chlamydomonas* sp.	4.1	132	*Ochromonas* CCMP1393	3.8	175
Microcystis sp.	1.7	170	Didiniidae	2.3	189	*Peridinium* sp. 1	3.1	62	*Chrysochromulina* CCMP291	2.3	102
Flavobacterium sp.	1.6	185	*Sphaeroeca leprechaunica*	2.3	190	*Tabellaria fenestrata*	3.0	155	Unclassified	2.0	133
GKS98	1.4	172	*Chrysosphaerella* sp.	2.0	133	*Dinobryon divergens*	3.0	80	*Cryptomonas ovata*	1.7	89
SH3-11	1.2	88	Unclassified	1.8	104	*Cryptomonas ovata*	2.9	155	Rhodophytes unclassified	1.5	60
Unclassified	1.1	112	Chrysophyceae cladeC	1.7	74	Unclassified flagellate	2.3	118	*Acanthoceras zachariasii*	1.3	75
Caulobacter sp.	0.9	128	Unclassified ochrophyta	1.6	36	*Cryptomonas* sp.	2.0	89	Unclassified	1.0	108
Babeliae unclassified	0.8	158	*Uroglena americana*	1.6	162	*Uronema* sp.	1.4	99	*Heterosigma akashimo*	1.0	175
Phenylobacterium sp.	0.7	133	Unclassified	1.6	42	Unclassified	1.0	81	*Synura* LO234KE	0.9	115
Polaromonas sp.	0.7	166	*Dinobryon bavaricum*	1.4	114	*Cryptomonas marssonii*	0.9	49	*Hemiselmis* sp.	0.9	152
IMCC26134	0.7	100	*Dinobryon* sp.	1.3	94	Cyanobacteria unclassified	0.9	144	*Mallomonas splendens*	0.6	133
Methylotenera sp.	0.7	196	*Askenasia* sp.	1.3	102	*Pediastrum tetras*	0.9	200	*Pseudopedinella elastica*	0.4	58
Aphanizomenon sp.	0.7	84	StrobilidiidaeB	1.1	181	*Aulacoseira* cf. *italica*	0.5	200	*Chromulina* sp.	0.4	98

Legend 16S rRNA
- Alphaproteobacteria
- Bacteroidetes
- Betaproteobacteria
- Cyanobacteria
- Gammaproteobacteria
- Planctomycetes
- Verrucomicrobia
- Others

Legend 18S rRNA, microscopy and chloroplast 16S rRNA
- Cercozoa
- Chlorophyta
- Ciliophora
- Cryptophyta
- Dinoflagellata
- Haptophyta
- Katablepharidophyta
- Telonemia
- Ochrophyta
- Others
- Unknown

Supplementary Table 7. Most abundant taxa (OTUs were pooled at their lowest taxonomic ranks) in Lake Saint-Charles for 16S rRNA, 18S rRNA, microscopy, and chloroplast 16S rRNA during the ice-cover period (Jan-Feb-Mar); %reads: mean relative abundance in % of total reads, Biovolume%: mean relative abundance in % of total biovolume, CV%: coefficient of variation, SD as % mean.

Lake Saint-Charles ice-cover											
16S rRNA			18S rRNA			Microscopy			Chloroplast 16S rRNA		
Taxonomy	Reads %	CV%	Taxonomy	Reads %	CV%	Taxonomy	Biovolume %	CV%	Taxonomy	Reads %	CV%
Unclassified	26.3	67	*Synura petersenii*	21.3	126	*Chlamydomonas* sp.	34.9	118	Unclassified	25.9	135
Unclassified	7.0	72	StrombidiidaA	9.9	50	*Strobilidium* sp.	27.6	89	*Cryptomonas erosa*	22.6	45
Unclassified	6.9	39	Cryptomonadales	6.9	118	*Cryptomonas ovata*	14.9	92	*Synura uvella*	19.0	79
Sediminibacterium sp.	4.6	66	Unclassified	4.6	61	*Cryptomonas marssonii*	5.6	173	*Teleaulax amphioxeia*	10.9	147
Methylobacter sp.	4.5	81	Protaspa lineage	4.2	110	*Uronema* sp.	5.1	97	Unclassified	6.7	120
Nitrosospira sp.	4.4	163	Chrysophyceae cladeE	3.9	126	*Peridinium* sp. 2	2.8	88	*Cryptomonas curvata*	5.0	91
Polaromonas sp.	4.0	155	Chrysophyceae	3.0	94	*Dinobryon divergens*	2.4	118	*Chrysochromulina* CCMP291	4.9	118
Unclassified	3.9	65	Chrysophyceae cladeD	2.8	68	*Oocystis* sp.	2.1	173	*Epipyxis* PR26KG	2.0	161
Unclassified	3.6	134	*Vorticella* sp.	2.8	81	*Asterionella formosa*	2.1	173	*Cryptomonas ovata*	1.4	87
Polynucleobacter sp.	3.3	63	Synura sp.	2.7	111	Unclassified flagellate 2	1.0	97	*Florenciella parvula*	0.8	66
Unclassified	2.4	68	*Cryptomonas curvata*	2.3	26	*Peridinium* sp. 1	0.7	173	*Guillardia theta*	0.2	60
Nitrotoga sp.	2.4	156	Chrysophyceae cladeF	2.3	80	*Chrysochromulina* sp.	0.6	173	*Dinobryon* LO226KS	0.2	85
SH3-11	1.8	72	Chrysophyceae 1	2.1	67	Picocyanobacteria	0.2	173	*Chroomonas caudata*	0.1	82
CL500-3	1.7	124	*Cryptomonas pyrenoidifera*	2.1	76				Rhodophytes unclassified	0.1	129
Luteolibacter sp.	1.5	173	*Chrysochromulina parva*	1.3	79				*Acanthoceras zachariasii*	0.0	88
Acidobacteria subgroup 3	1.5	129	Choreotrichida	1.3	79				*Chromulina* sp.	0.0	138
Actinobacteria unclassified	1.4	64	Chrysophyceae cladeC	1.2	99				*Pseudopedinella elastica*	0.0	132
Rhodovastum sp.	1.3	97	Litostomatea	1.0	164				*Chrysosphaera* sp.	0.0	100
Margulisbacteria unclassified	1.3	84	Unclassified	1.0	49				Unclassified	0.0	100
Flavobacterium sp.	1.0	50	*Askenasia* sp.	0.9	68				*Hemiselmis* sp.	0.0	87

Legend 16S rRNA
- Alphaproteobacteria
- Bacteroidetes
- Betaproteobacteria
- Cyanobacteria
- Gammaproteobacteria
- Planctomycetes
- Verrucomicrobia
- Others

Legend 18S rRNA, microscopy and chloroplast 16S rRNA
- Cercozoa
- Chlorophyta
- Ciliophora
- Cryptophyta
- Dinoflagellata
- Haptophyta
- Katablepharidophyta
- Telonemia
- Ochrophyta
- Others
- Unknown

Supplementary Table 8. Most abundant taxa (OTUs were pooled at their lowest taxonomic ranks) in Lake Clément for 16S rRNA, 18S rRNA, microscopy, and chloroplast 16S rRNA during the open-water period; %reads: mean relative abundance in % of total reads, Biovolume%: mean relative abundance in % of total biovolume, CV%: coefficient of variation, SD as % mean.

Lake Clément open water

16S rRNA

Taxonomy	Reads %	CV%
Unclassified	28.62	41
Unclassified	21.12	26
Polynucleobacter sp.	5.21	72
CL500-3	5.05	94
Unclassified	3.69	38
Unclassified	2.99	140
Dinghuibacter sp.	2.34	73
SH3-11	2.27	56
Actinobacteria unclassified	2.21	85
Fimbriimonadia unclassified	1.99	111
Sediminibacterium sp.	1.68	74
Actinobacteria hgcI clade	1.53	44
Deltaproteobacteria unclassified	1.42	150
Deltaproteobacteria OM27 clade	1.38	141
MWH-UniP1	1.35	171
Flavobacterium sp.	1.10	137
Polaromonas sp.	1.05	66
Unclassified	0.89	20
Hirschia sp.	0.75	39
Fluviicola sp.	0.71	44

18S rRNA

Taxonomy	Reads %	CV%
Litostomatea	10.79	65
Dinobryon divergens	10.61	142
Chrysophyceae cladeC	8.05	22
Dinobryon bavaricum	6.29	144
StrombidiidaA	5.94	42
Halteriidae	5.73	82
Cryptomonadales	4.90	48
Mallomonas caudata	4.72	173
Chrysophyceae	4.71	21
Chrysophyceae B1	3.41	6
Diacronema sp.	2.79	103
Cryptophyceae 1	2.49	81
Chlamydomonadales	2.36	75
Cryptomonas curvata	2.10	104
Hypotrichia	2.01	51
Katablepharidales	1.97	105
Novel clade 2	1.65	109
Chrysochromulina parva	1.62	100
Plagioselmis sp.	1.86	56
Mallomonas sp.	1.52	99

Microscopy

Taxonomy	Biovolume %	CV%
Chrysochromulina sp.	55.35	88
Strobilidium sp.	15.97	73
Dinobryon divergens	6.89	101
Cryptomonas marssonii	6.64	79
Ceratium hirundinella	4.61	173
Cryptomonas ovata	1.66	155
Uronema sp.	1.48	173
Oocystis sp.	1.15	173
Cryptomonas sp.	1.15	173
Unclassified	1.13	104
Chloromonas sp.	0.54	109
Chlamydomonas sp.	0.43	141
Mallomonas caudata	0.37	58
Unclassified flagellate	0.33	173
Unclassified flagellate 2	0.19	90
Asterococcus sp.	0.06	88
Elakatothrix sp.	0.04	173
Kephyrion sp.	0.04	173
Pseudanabaena sp.	0.02	173
Ophiocytium sp.	0.01	173

Chloroplast 16S rRNA

Taxonomy	Reads %	CV%
Dinobryon LO226KS	21.93	137
Synura uvella	12.48	72
Teleaulax amphioxeia	10.96	80
Cryptomonas erosa	10.27	36
Florenciella parvula	10.02	65
Unclassified	7.14	90
Chrysophytes unclassified	6.62	47
Epipyxis PR26KG	5.61	92
Chrysochromulina CCMP291	4.16	83
Rhodophytes unclassified	3.55	51
Pavlova gyrans	1.89	115
Ochromonas CCMP1393	0.65	88
Oophila amblystomatis	0.63	70
Pseudopedinella elastica	0.56	114
Chlamydomonas asymmetrica	0.38	83
Unclassified	0.34	111
Cryptomonas curvata	0.27	86
Hemiselmis sp.	0.25	25
Virgulinella fragilis	0.15	94
Guillardia theta	0.02	115

Legend 16S rRNA

- Alphaproteobacteria
- Bacteroidetes
- Betaproteobacteria
- Cyanobacteria
- Gammaproteobacteria
- Planctomycetes
- Verrucomicrobia
- Others

Legend 18S rRNA, microscopy and chloroplast 16S rRNA

- Cercozoa
- Chlorophyta
- Ciliophora
- Cryptophyta
- Dinoflagellata
- Haptophyta
- Katablepharidophyta
- Telonemia
- Ochrophyta
- Others
- Unknown

Supplementary Table 9. Most abundant taxa (OTUs were pooled at their lowest taxonomic ranks) in Lake Clément for 16S rRNA, 18S rRNA, microscopy, and chloroplast 16S rRNA during the ice-cover period (Jan-Feb-Mar); %reads: mean relative abundance in % of total reads, Biovolume%: mean relative abundance in % of total biovolume, CV%: coefficient of variation, SD as % mean.

Lake Clément ice-cover

16S rRNA

Taxonomy	Reads %	CV%
Unclassified	19.8	15
Unclassified	16.1	16
Unclassified	13.1	26
Unclassified	8.0	65
Methylobacter sp.	7.6	53
CL500-3	6.4	91
Unclassified	4.2	13
Polaromonas sp.	2.8	52
Polynucleobacter sp.	2.6	33
Sediminibacterium sp.	2.0	40
Unclassified	1.3	54
CM1G08	1.2	46
Fluviicola sp.	1.2	68
SH3-11	1.1	114
Actinobacteria hgcI clade	0.9	38
Actinobacteria unclassified	0.9	38
Nitrosospira sp.	0.8	148
IMCC26134	0.7	133
Methylotenera sp.	0.7	40
Rhodoferax sp.	0.5	78

18S rRNA

Taxonomy	Reads %	CV%
Cryptomonas sp.	28.1	89
Chrysophyceae cladeF	16.1	49
Novel clade 10	9.3	86
Halteria grandinella	7.7	162
Group 2	7.4	117
Cryptophyceae 1	4.8	82
Cryptomonadales	4.4	118
Borghiella tenuissima	3.7	171
Chrysophyceae cladeD	3.7	115
Chrysophyceae	2.7	97
Parmales	1.3	42
StrombidiidaA	1.1	87
Chrysophyceae cladeC	1.0	99
Ochrophyta	0.9	158
Halteriidae	0.9	103
Cryptomonas curvata	0.7	107
Chlamydomonadales	0.6	167
Protaspa lineage	0.5	132
Plagioselmis sp.	0.5	161
Philasterida	0.4	103

Microscopy

Taxonomy	Biovolume %	CV%
Unclassified	50.5	115
Uronema sp.	16.3	173
Picocyanobacteria	12.0	173
Cryptomonas sp.	8.0	173
Peridinium sp. 2	4.3	62
Chloromonas sp.	2.9	173
Unclassified choanoflagellate	1.9	173
Dinobryon divergens	1.2	173
Chrysochromulina sp.	1.1	173
Unclassified flagellate 2	0.9	87
Cryptomonas marssonii	0.8	173
Ophiocytium sp.	0.5	173
Chlamydomonas sp.	0.2	173
Cymbella sp.	0.1	173
Synedra sp.	0.1	173

Chloroplast 16S rRNA

Taxonomy	Reads %	CV %
Cryptomonas erosa	78.7	83
Unclassified	8.7	86
Teleaulax amphioxeia	4.3	137
Epipyxis PR26KG	4.2	173
Chrysophytes unclassified	1.9	47
Florenciella parvula	1.1	80
Cryptomonas curvata	0.4	121
Pavlova gyrans	0.1	122
Synura uvella	0.1	20
Chrysochromulina CCMP291	0.1	66
Dinobryon LO226KS	0.0	120
Chrysosphaera sp.	0.0	132
Hemiselmis sp.	0.0	115
Guillardia theta	0.0	173
Ochromonas CCMP1393	0.0	0
Pseudopedinella elastica	0.0	173
Oophila amblystomatis	0.0	173
Skeletonema pseudocostatum	0.0	173

Legend 16S rRNA

- Alphaproteobacteria
- Bacteroidetes
- Betaproteobacteria
- Cyanobacteria
- Gammaproteobacteria
- Planctomycetes
- Verrucomicrobia
- Others

Legend 18S rRNA, microscopy and chloroplast 16S rRNA

- Cercozoa
- Chlorophyta
- Ciliophora
- Cryptophyta
- Dinoflagellata
- Haptophyta
- Katablepharidophyta
- Telonemia
- Ochrophyta
- Others
- Unknown

Supplementary Table 10. Most abundant taxa (OTUs were pooled at their lowest taxonomic ranks) in Lake Saint-Augustin for 16S rRNA, 18S rRNA, microscopy, and chloroplast 16S rRNA during the open-water period; %reads: mean relative abundance in % of total reads, Biovolume%: mean relative abundance in % of total biovolume, CV%: coefficient of variation, SD as % mean.

Lake Saint-Augustin open water											
16S rRNA			18S rRNA			Microscopy			Chloroplast 16S rRNA		
Taxonomy	Reads %	CV %	Taxonomy	Reads %	CV %	Taxonomy	Biovolume %	CV %	Taxonomy	Reads %	CV %
CL500-3	15.2	50	Litostomatea	17.8	40	*Aphanocapsa* sp.	31.5	173	*Chrysochromulina* CCMP291	28.7	52
Unclassified	14.0	13	*Chrysochromulina parva*	7.4	85	*Peridinium* sp. 1	18.0	66	*Cryptomonas curvata*	25.0	133
Unclassified	12.9	61	CONTH4 unclassified	6.8	122	*Chrysochromulina* sp.	16.3	85	*Cryptomonas erosa*	22.7	36
Unclassified	8.0	73	*Gymnodinium* sp.	6.3	116	*Strobilidium* sp.	6.2	68	*Teleaulax amphioxeia*	12.2	89
Cuspidothrix sp.	4.7	91	*Obertrumia georgiana*	5.8	173	*Chrysochromulina parva*	5.4	169	Chrysophytes unclassified	3.8	109
Unclassified	4.2	75	*Cryptomonas* sp.	5.5	110	*Asterionella formosa*	5.0	173	*Florenciella parvula*	2.2	93
Polynucleobacter sp.	3.0	20	Katablepharidales	4.7	70	*Peridinium* sp. 2	4.9	129	*Synura uvella*	1.9	165
Unclassified	2.7	106	CONThreeP unclassified	4.1	130	*Ceratium furca*	4.0	173	*Epipyxis* PR26KG	1.8	162
SH3-11	2.6	109	Cryptophyceae 1	3.4	132	*Cryptomonas ovata*	4.0	58	Unclassified	0.8	82
Flavobacterium sp.	2.2	153	*Epistylis* sp.	3.3	173	*Cryptomonas* sp.	2.5	173	*Acanthoceras zachariasii*	0.4	169
Cyanobium sp.	2.0	167	*Askenasia* sp.	3.2	145	*Tabellaria fenestrata*	2.3	173	*Skeletonema pseudocostatum*	0.3	129
Sediminibacterium sp.	1.6	162	*Tintinnidium fluviatile*	2.7	167	*Chlamydomonas* sp.	1.7	126	*Pseudopedinella elastica*	0.1	102
Deltaproteobacteria unclassified	1.6	82	StrombidiidaA	2.6	161	*Pediastrum tetras*	1.7	108	*Chroomonas caudata*	0.0	125
Unclassified	1.6	87	*Vorticella microstoma*	1.9	112	*Cryptomonas marssonii*	1.6	132	Unclassified	0.0	173
Unclassified	1.2	64	Thoracosphaeraceae	1.9	173	*Aphanizomenon flos-aquae*	1.5	173	*Chrysosphaera* sp.	0.0	173
Dolichospermum sp.	1.1	85	Pseudoholophrya	1.5	83	*Dolichospermum planctonicum*	0.8	94	*Guillardia theta*	0.0	173
MWH-UniP1	1.1	125	Unclassified	1.4	127	cf. *Glaucoma scintillans*	0.7	173			
Reyranella sp.	1.0	158	*Cryptomonas tetrapyrenoidosa*	1.3	101	*Uronema* sp.	0.6	131			
Fluviicola sp.	0.7	73	CONTH5 unclassified	1.2	116	*Trachelomonas* sp. 1	0.5	173			
Dinghuibacter sp.	0.6	59	Novel clade 10	1.1	14	*Asterococcus* sp.	0.2	173			

Legend 16S rRNA
- Alphaproteobacteria
- Bacteroidetes
- Betaproteobacteria
- Cyanobacteria
- Gammaproteobacteria
- Planctomycetes
- Verrucomicrobia
- Others

Legend 18S rRNA, microscopy and chloroplast 16S rRNA
- Cercozoa
- Chlorophyta
- Ciliophora
- Cryptophyta
- Dinoflagellata
- Haptophyta
- Katablepharidophyta
- Telonemia
- Ochrophyta
- Others
- Unknown

Supplementary Table 11. Most abundant taxa (OTUs were pooled at their lowest taxonomic ranks) in Lake Saint-Augustin for 16S rRNA, 18S rRNA, microscopy, and chloroplast 16S rRNA during the ice-cover period (Jan-Feb-Mar); %reads: mean relative abundance in % of total reads, Biovolume%: mean relative abundance in % of total biovolume, CV%: coefficient of variation, SD as % mean.

Lake Saint-Augustin ice-cover

16S rRNA

Taxonomy	Reads %	CV%
CL500-3	21.5	52
Unclassified	18.5	12
Unclassified	9.3	75
Flavobacterium sp.	7.6	130
Undibacterium sp.	7.4	139
Polaromonas sp.	4.0	117
Unclassified	3.7	78
Unclassified	3.6	83
Deltaproteobacteria OM27 clade	2.7	91
Aquirestis sp.	1.6	130
Unclassified	1.6	4
Dinghuibacter sp.	1.5	83
Unclassified	1.0	78
Terrimicrobium sp.	1.0	155
Polynucleobacter sp.	0.9	54
Actinobacteria hgcI clade	0.9	78
Rhodoferax sp.	0.8	92
Methylotenera sp.	0.7	166
Actinobacteria unclassified	0.7	65
Nitrosospira sp.	0.7	111

18S rRNA

Taxonomy	Reads %	CV%
Chlamydomonadales	15.7	129
Chrysophyceae cladeF	11.0	104
Chrysophyceae	9.9	84
Cryptomonadales	8.8	80
Chrysophyceae cladeC	6.7	172
Cryptomonas curvata	5.9	55
Chrysophyceae cladeD	4.9	79
Plagioselmis sp.	4.3	113
Cryptophyceae 1	3.6	73
Katablepharidales	2.6	95
Stephanodiscus sp.	2.3	118
Prorocentrum sp.	2.1	158
Chrysochromulina parva	2.1	82
Unclassified	2.0	116
Spumella vulgaris	1.8	173
Protaspa lineage	1.5	16
Parmales	1.4	71
Philasterida	0.7	60
Rimostrombidium sp.	0.7	166
Askenasia sp.	0.6	108

Microscopy

Taxonomy	Biovolume %	CV%
Chrysochromulina parva	26.2	106
Chlamydomonas sp.	23.4	111
Tabellaria fenestrata	17.5	173
Cryptomonas ovata	15.9	89
Unclassified	6.4	116
Peridinium sp. 2	5.7	76
Trachelomonas sp. 2	1.3	173
Trachelomonas sp. 1	1.2	124
Uronema sp.	0.9	173
Cryptomonas marssonii	0.8	173
Chrysochromulina sp.	0.3	173
Borghiella sp.	0.2	173
Unclassified flagellate 2	0.2	173
Ophiocytium sp.	0.0	173

Chloroplast 16S rRNA

Taxonomy	Reads %	CV%
Cryptomonas erosa	38.1	59
Teleaulax amphioxeia	25.0	87
Unclassified	16.1	79
Chrysochromulina CCMP291	8.3	101
Florenciella parvula	3.4	97
Skeletonema pseudocostatum	2.5	108
Cryptomonas curvata	1.1	135
Epipyxis PR26KG	0.7	140
Chromulina sp.	0.7	109
Ochromonas CCMP1393	0.4	168
Oophila amblystomatis	0.4	80
Chrysophytes unclassified	0.3	71
Chrysosphaera sp.	0.1	48
Dinobryon LO226KS	0.0	173
Synura uvella	0.0	173
Unclassified	0.0	173
Guillardia theta	0.0	173

Legend 16S rRNA

- Alphaproteobacteria
- Bacteroidetes
- Betaproteobacteria
- Cyanobacteria
- Gammaproteobacteria
- Planctomycetes
- Verrucomicrobia
- Others

Legend 18S rRNA, microscopy and chloroplast 16S rRNA

- Cercozoa
- Chlorophyta
- Ciliophora
- Cryptophyta
- Dinoflagellata
- Haptophyta
- Katablepharidophyta
- Telonemia
- Ochrophyta
- Others
- Unknown

Supplementary Table 12. Taxa identified by the DESeq analysis to discriminate between the lakes with the lower conductivity/urbanization (Lake Clair and Lake Saint-Charles) and the lakes with the higher conductivity/urbanization (Lake Clément and Lake Saint-Augustin) for 16S rRNA, 18S rRNA, microscopy and chloroplast 16S rRNA. For the data resulting from the rRNA analysis, the OTUs were pooled at their lowest taxonomic ranks. The DESeq analysis was conducted on the two periods (ice-cover and open-water) combined. L2FC: Average log^2 fold change between the two groups of lakes. CV%: coefficient of variation, SD as % mean. Low: mean relative (%) abundance (in reads for 16S, 18S and chloroplasts 16S) or biovolume (microscopy) in the lakes with the lower conductivity/urbanization. Taxa with a higher relative abundance (in reads) or biovolume in this group of lakes are in **bold**. High: mean relative (%) abundance (in reads for 16S, 18S and chloroplasts 16S) or biovolume (microscopy) in the lakes with the higher conductivity/urbanization.

16S rRNA

Taxonomy	L2FC	Low	High
***Chthoniobacter* sp.**	**7.2**	**0.9**	**0.7**
**Unclas.**	**6.6**	**0.9**	**0.6**
**Soil bacterium**	**5.9**	**0.4**	**<0.1**
**Unclas.**	**5.8**	**0.2**	**0.1**
***Phenylobacterium* sp.**	**5.6**	**0.6**	**<0.1**
***Chthoniobacter* sp.**	**5.5**	**1.0**	**0.8**
**Unclas.**	**5.5**	**1.0**	**<0.1**
***Dinghuibacter* sp.**	**5.3**	**0.4**	**<0.1**
**Unclas.**	**5.1**	**0.3**	**<0.1**
**Unclas.**	**5.0**	**0.2**	**0.1**
CL500-3	-6.6	<0.1	1.2
Algoriphagus hongiella	-6.6	<0.1	0.3
CL500-3	-6.3	<0.1	0.6
CL500-3	-6.1	<0.1	0.7
Unclas.	-5.9	<0.1	0.5
MWH-UniP1	-5.8	<0.1	0.3
Unclas.	-5.8	<0.1	0.3
Cuspdothrix LMECYA-163	-5.8	<0.1	0.8
OM190	-5.7	<0.1	0.6
Fimbriimonadia unclas.	-5.5	<0.1	<0.1

18S rRNA

Taxonomy	L2FC	Low	High
***Mesodinium* sp.**	**8.4**	**7.6**	**<0.1**
***Mesodinium* sp.**	**7.0**	**3.4**	**0.2**
***Mesodinium* sp.**	**6.8**	**2.6**	**<0.1**
***Mesodinium* sp.**	**5.8**	**1.3**	**0.1**
***Mesodinium* sp.**	**5.4**	**3.8**	**0.1**
Dinobryon sociale	**4.7**	**0.6**	**<0.1**
**StrobilidiidaeH**	**4.5**	**0.3**	**<0.1**
***Mesodinium* sp.**	**4.5**	**2.0**	**0.4**
***Mesodinium* sp.**	**4.4**	**1.3**	**<0.1**
**StrombidiidaA**	**4.2**	**0.4**	**<0.1**
Plagioselmis sp.	-5.2	<0.1	1.4
StrombidiidaA	-5.1	<0.1	1.9
Cryptomonas sp.	-4.9	<0.1	7.5
Katablepharidales	-4.6	<0.1	0.9
Cryptomonadales	-4.3	<0.1	0.6
Katablepharidales	-4.0	<0.1	0.6
Cryptomonas sp.	-4.0	<0.1	0.8
Diacronema sp.	-4.0	<0.1	0.7
Diacronema sp.	-3.8	<0.1	0.5
Litostomatea	-3.5	<0.1	0.5

Microscopy

Taxonomy	L2FC	Low	High
Dinobryon bavaricum	**15.0**	**10.5**	**<0.1**
Synura uvella	**7.5**	**4.3**	**<0.1**
***Eutreptia* sp.**	**7.4**	**0.0**	**<0.1**
***Rabdoderma* sp.**	**6.4**	**0.2**	**<0.1**
**Cyanobacteria unclas.**	**6.0**	**4.1**	**<0.1**
***Urosolenia* sp.**	**5.4**	**0.1**	**<0.1**
***Kephyrion* sp.**	**4.9**	**<0.1**	**<0.1**
Dinobryon petiolatum	**4.8**	**<0.1**	**<0.1**
***Crucigenia* sp.**	**4.5**	**<0.1**	**<0.1**
***Cyclotella* sp.**	**4.5**	**<0.1**	**<0.1**
***Merismopedia* sp.**	**2.5**	**0.1**	**<0.1**
***Chroococcus* sp.**	**2.3**	**<0.1**	**<0.1**
Dolichospermum planctonicum	-11.8	<0.1	0.4
Chloromonas sp.	-8.8	0.1	1.0
cf. *Polytomella* sp.	-6.9	0.4	0.5
Peridinium sp. 2	-5.7	0.8	6.1
Ochrophyte unclas.	-5.5	5.9	13.2
Choanoflagellate unclas.	-4.0	<0.1	0.5
Ophiocytium sp.	-3.3	<0.1	0.2

Chloroplast 16S rRNA

Taxonomy	L2FC	Low	High
Synura uvella	**5.5**	**6.9**	**<0.1**
Cryptomonas ovata	**4.4**	**0.9**	**<0.1**
Synura uvella	**3.2**	**14.8**	**14.8**
Synura uvella	**2.8**	**1.3**	**<0.1**
**Rhodophytes unclas.**	**2.8**	**0.5**	**<0.1**
**Unclas.**	**2.3**	**4.6**	**1.7**
**Unclas.**	**2.3**	**2.6**	**0.2**
***Epipyxis* PR26KG**	**2.2**	**0.4**	**<0.1**
**Unclas.**	**2.2**	**0.5**	**<0.1**
***Synura* LO234KE**	**2.1**	**0.5**	**<0.1**
Cryptomonas curvata	-6.8	<0.1	3.2
Pavlova gyrans	-5.4	<0.1	0.1
Cryptomonas erosa	-5.2	0.1	16.4
Teleaulax amphioxeia	-5.0	0.1	0.9
Teleaulax amphioxeia	-5.0	1.8	5.7
Cryptomonas curvata	-4.8	<0.1	0.6
Teleaulax amphioxeia	-4.8	0.5	2.8
Skeletonema pseudocastatum	-4.7	0.1	0.6
Rhodophytes unclas.	-4.2	<0.1	<0.1
Chrysochromulina CCMP291	-3.6	<0.1	0.1

Legend 16S rRNA

- Alphaproteobacteria
- Bacteroidetes
- Betaproteobacteria
- Cyanobacteria
- Gammaproteobacteria
- Planctomycetes
- Verrucomicrobia
- Others

Legend 18S rRNA, microscopy and chloroplast 16S rRNA

- Cercozoa
- Chlorophyta
- Ciliophora
- Cryptophyta
- Dinoflagellata
- Haptophyta
- Katablepharidophyta
- Telonemia
- Ochrophyta
- Others
- Unknown

Supplementary Table 13. Taxa dentified by the DESeq analysis to discriminate between the ice-cover (Jan-Feb-Mar) and the open-water (rest of the year) periods for 16S rRNA, 18S rRNA, microscopy and chloroplasts 16S rRNA. For the data resulting from the rRNA analysis, the OTUs were pooled at their lowest taxonomic ranks. The DESeq analysis was conducted on the four lakes combined. L2FC: Average log^2 fold change between the two periods. CV%: coefficient of variation, SD as % mean. IC: mean relative (%) abundance (in reads for 16S, 18S and chloroplast 16S) or biovolume (microscopy) during the ice-cover period. Taxa with a higher relative abundance (in reads) or biovolume in this group of lakes are in **bold**. OW: mean relative (%) abundance (in reads for 16S, 18S and chloroplast 16S) or biovolume (microscopy) during the open-water period.

16S rRNA

Taxonomy	L2FC	IC	OW
***Nitrosospira* sp.**	**7.8**	**1.0**	**<0.1**
**Unclas.**	**7.1**	**1.3**	**<0.1**
***Flavobacterium* sp.**	**7.0**	**1.0**	**<0.1**
***Methylobacter* sp.**	**6.2**	**2.5**	**<0.1**
**Unclas.**	**6.1**	**0.7**	**<0.1**
**Unclas.**	**6.0**	**1.6**	**<0.1**
***Nitrotoga* sp.**	**5.0**	**0.4**	**<0.1**
**CL500-3**	**5.0**	**0.8**	**<0.1**
**Unclas.**	**4.9**	**1.0**	**<0.1**
**Subgroup 6**	**4.7**	**0.1**	**<0.1**
Unclas.	-7.0	<0.1	0.7
Unclas.	-6.1	<0.1	0.4
CL500-3	-5.8	<0.1	0.8
Deltaproteobacteria unclas.	-5.7	<0.1	0.3
Unclas.	-5.5	<0.1	0.3
Unclas.	-5.3	<0.1	0.2
Unclas.	-5.2	<0.1	0.2
Unclas.	-5.2	<0.1	0.3
Dinghuibacter sp.	-4.9	<0.1	0.5
Rhizorhapis sp.	-4.8	<0.1	0.1

18S rRNA

Taxonomy	L2FC	IC	OW
Synura mollispina	**6.3**	**8.5**	**<0.1**
**Chrysophyceae_cladeF**	**6.3**	**4.7**	**<0.1**
**StrombidiidaA**	**5.9**	**2.1**	**<0.1**
Halteria grandinella	**5.9**	**1.1**	**<0.1**
**Chrysophyceae_cladeF**	**5.6**	**0.8**	**<0.1**
Halteria grandinella	**5.2**	**0.6**	**<0.1**
Synura petersenii	**5.0**	**3.7**	**<0.1**
**Chrysophyceae_cladeD**	**4.8**	**0.6**	**<0.1**
**Chrysophyceae_cladeC**	**4.5**	**0.4**	**<0.1**
**Chrysophyceae_cladeD**	**4.4**	**0.3**	**<0.1**
Dinobryon divergens	-6.3	<0.1	2.0
Litostomatea	-6.2	<0.1	1.7
Dinobryon bavaricum	-5.3	<0.1	1.2
Litostomatea	-4.8	<0.1	0.6
Litostomatea	-4.8	<0.1	0.6
Litostomatea	-4.8	<0.1	0.6
CONThreeP_uncl	-4.4	<0.1	0.6
Chrysophyceae_cladeC	-4.4	<0.1	0.5
CONThreeP_uncl	-4.4	<0.1	0.5
Litostomatea	-4.2	<0.1	0.4

Microscopy

Taxonomy	L2FC	IC	OW
**cf. *Polytomella* sp.**	**7.1**	**0.8**	**0.1**
***Chloromonas* sp.**	**5.7**	**0.9**	**0.1**
Pediastrum tetras	-13.9	<0.1	0.9
Ochrophyte unclas.	-13.7	0.3	18.7
Unclassified flagellate 1	-11.9	<0.1	0.5
Mallomonas tonsurata	-10.6	<0.1	0.1
Bitrichia sp.	-9.1	<0.1	<0.1
Peridinium sp. 1	-8.0	0.3	3.5
Scenedesmus sp.	-7.8	<0.1	<0.1
Unclas.	-7.5	<0.1	0.2
Kephyrion sp.	-7.3	<0.1	<0.1
Synura uvella	-7.1	<0.1	4.3
Rabdoderma sp.	-6.0	<0.1	0.2
Cyanobacteria unclas.	-5.6	<0.1	4.1
Urosolenia sp.	-5.0	<0.1	0.1
Choanoflagellate unclas.	-4.7	0.5	0.1
Picocyanobacteria unclas.	-4.6	3.0	5.6
Kephyrion sp. 2	-4.5	<0.1	<0.1
Dinobryon petiolatum	-4.4	<0.1	<0.1
Strobilidium sp.	-4.1	8.1	12.2

Chloroplast 16S rRNA

Taxonomy	L2FC	IC	OW
Synura uvella	**8.5**	**14.8**	**<0.1**
***Epipyxis* PR26KG**	**7.1**	**1.4**	**<0.1**
Florenciella parvula	**6.8**	**1.7**	**<0.1**
Synura uvella	**4.7**	**6.8**	**0.1**
Cryptomonas erosa	**4.4**	**16.5**	**0.2**
Teleaulax amphioxeia	**4.4**	**5.4**	**4.2**
Cryptomonas erosa	**4.2**	**6.8**	**1.1**
**Unclas.**	**3.9**	**12.3**	**2.4**
Teleaulax amphioxeia	**3.4**	**2.5**	**1.5**
***Chromulina* sp.**	**3.0**	**0.1**	**<0.1**
Synura uvella	-5.2	<0.1	1.8
Epipyxis PR26KG	-4.6	<0.1	1.4
Dinobryon LO226KS	-4.3	<0.1	1.2
Rhodophytes unclas.	-4.2	<0.1	0.9
Epipyxis PR26KG	-3.7	0.2	3.0
Epipyxis PR26KG	-3.6	<0.1	0.8
Synura uvella	-3.6	<0.1	0.5
Hemiselmis tepida	-3.4	<0.1	0.7
Unclas.	-3.1	<0.1	0.6
Synura uvella	-2.9	<0.1	1.3

Legend 16S rRNA

- Alphaproteobacteria
- Bacteroidetes
- Betaproteobacteria
- Cyanobacteria
- Gammaproteobacteria
- Planctomycetes
- Verrucomicrobia
- Others

Legend 18S rRNA, microscopy and chloroplast 16S rRNA

- Cercozoa
- Chlorophyta
- Ciliophora
- Cryptophyta
- Dinoflagellata
- Haptophyta
- Katablepharidophyta
- Telonemia
- Ochrophyta
- Others
- Unknown
